# A Creatinine–CAR Composite Index (CCAR) Optimized by Machine Learning for Prognosis in Cancer Cachexia

**DOI:** 10.1002/jcsm.70120

**Published:** 2025-11-11

**Authors:** Heyang Zhang, Chong Li, Qiuyi Chen, Jinyu Shi, Wenjing Wang, Guotian Ruan, Chenan Liu, Shutian Zhang, Shengtao Zhu, Peng Li, Hanping Shi

**Affiliations:** ^1^ Department of Gastroenterology, Beijing Friendship Hospital Capital Medical University, National Clinical Research Center for Digestive Diseases, Beijing Digestive Disease Center, Beijing Key Laboratory for Precancerous Lesion of Digestive Diseases Beijing China; ^2^ Department of Gastroenterology, Beijing Friendship Hospital Capital Medical University Beijing China; ^3^ State Key Laboratory for Digestive Health Beijing China; ^4^ National Clinical Research Center for Digestive Diseases Beijing China; ^5^ Department of Oncology The Affiliated Dazu's Hospital of Chongqing Medical University Chongqing China; ^6^ Department of Pharmacology, School of Basic Medical Sciences Anhui Medical University Hefei China; ^7^ Key Laboratory of Cancer FSMP for State Market Regulation Beijing China

**Keywords:** Cachexia, creatinine–CAR composite index, machine learning, web‐based calculator

## Abstract

**Background:**

Cancer cachexia is a multifactorial syndrome associated with poor prognosis and impaired quality of life in cancer patients. However, survival prediction in cancer cachexia remains difficult due to the lack of reliable biomarkers.

**Methods:**

This retrospective cohort study analysed data from 1,367 patients with cancer cachexia diagnosed according to the 2011 Fearon consensus, using the multicentre INSCOC database. The cohort was divided into a training set (*n* = 959), an internal validation set (*n* = 408), and an independent external validation cohort (*n* = 284). The mean age of the entire cohort was 58.7 ± 10.9 years, and 39.4% were female. LASSO regression identified creatinine (Cr) as a key predictor. The CCAR (Cr + CAR) index was then constructed using Cr and CAR within a random forest model. Prognostic performance was assessed by Harrell's concordance index (C‐index), time‐dependent AUC, Kaplan–Meier analysis and multivariate Cox regression, with overall survival (OS) as the primary endpoint.

**Results:**

The CCAR index consistently outperformed conventional inflammation‐ and nutrition‐related markers across all three cohorts. The C‐index values for CCAR were 0.777 in the training cohort, 0.789 in the internal validation cohort, and 0.765 in the external validation cohort, compared with 0.627–0.660 for CAR ALONE. Patients in the high‐CCAR group had significantly worse OS than those in the low‐CCAR group (log‐rank *p* < 0.0001 for all cohorts). Multivariate Cox regression confirmed that CCAR was an independent prognostic factor for OS (HR 3.31, 95% CI 2.94–3.72 in the training set; HR 3.51, 95% CI 2.93–4.22 in the validation set; HR 3.21, 95% CI 2.55–4.03 in the external cohort; all *p* < 0.001). A web‐based calculator (https://heyangzhang.pythonanywhere.com) was developed for real‐time CCAR computation and survival predictions.

**Conclusion:**

The CCAR index provides a robust, easily accessible tool for predicting survival outcomes in cancer cachexia patients. The web‐based CCAR calculator demonstrates significant clinical applicability and improves patient risk stratification, offering potential for guiding early interventions and personalized treatment strategies in clinical settings.

## 1 Introduction

Cachexia is a complex and multifactorial syndrome commonly observed in patients with advanced cancer [1]. It is characterized by significant weight loss, muscle wasting and functional decline, often accompanied by severe metabolic and nutritional disturbances [2, 3]. The development of cachexia is driven by a combination of systemic inflammation, altered nutrient metabolism and the loss of muscle mass [4, 5, 6]. As a result, patients with cachexia experience not only diminished physical strength and quality of life but also poorer responses to cancer treatments and decreased survival rates. The interplay between inflammation and muscle degradation is central to the progression of cachexia, making it a critical area of focus for clinical research [7, 8]. Despite its clinical relevance, cachexia is often underdiagnosed and undertreated in routine oncology practice.

Several biomarkers have been proposed to predict the outcomes of cancer cachexia, including inflammatory markers such as C‐reactive protein (CRP), lymphocyte‐to‐CRP ratio (LCR) and the CRP‐to‐albumin ratio (CAR) [9, 10, 11]. These indicators are commonly used to assess the level of systemic inflammation and nutritional status in patients. However, while these markers provide valuable insights, their predictive power as standalone tools remains limited. Factors such as variability in patient populations, the multifactorial nature of cachexia, and the inability of single biomarkers to capture the complexity of cachexia all contribute to the relatively low accuracy of these predictions. Consequently, there is a growing need for more comprehensive models that combine multiple biomarkers to improve prognostic accuracy.

Despite the growing availability of prognostic tools, few models have integrated inflammation, nutrition and metabolic factors into a single predictive index. In our study, through Lasso regression, we identified creatinine (Cr) as a significant predictor in cancer cachexia patients, ranking highly among the variables. This suggested that Cr may be a key variable associated with cachexia. Therefore, we further explored the predictive power of various inflammatory markers in cachexia patients and found that the CRP‐to‐albumin ratio (CAR) performed the best among these composite indices. Based on this finding, we combined Cr and CAR to create a novel composite index, the creatinine‐C‐reactive protein‐to‐albumin ratio (CCAR), using random forest modelling. To enhance clinical applicability, we developed a user‐friendly CCAR calculator, which allows clinicians to input patient data, including Cr, albumin and CRP, and calculate the corresponding CCAR value, offering personalized survival predictions. This study specifically focuses on prognostic assessment in patients who have already been diagnosed with cancer cachexia.

## 2 Materials and Methods

### 2.1 Study Design and Patient Selection

This study was a retrospective cohort study conducted using the ‘Investigation on Nutritional Status and its Clinical Outcomes of Common Cancers’ (INSCOC) database. The INSCOC project, which is registered at chictr.org.cn (registration number ChiCTR1800020329), prospectively collects clinical data from cancer patients at multiple centers across China. This study focused on cancer patients with cachexia, and we initially identified 1,367 patients diagnosed with cachexia from this database. Cachexia was defined as meeting at least one of the following conditions: (1) unintentional weight loss > 5% within the previous 6 months; (2) body mass index (BMI) < 20 kg/m^2^ with additional weight loss > 2%; or (3) sarcopenia accompanied by weight loss > 2% within the past 6 months. Inclusion criteria: (1) patients diagnosed with solid tumours, including lung cancer, oesophageal cancer, gastric cancer, hepatobiliary malignancies (such as liver cancer and cholangiocarcinoma), pancreatic cancer, colorectal cancer, urological cancers, nasopharyngeal cancer, breast cancer and gynaecological cancers (including endometrial cancer, ovarian cancer and cervical cancer); (2) age over 18 years; (3) patients who have undergone detailed body composition measurements. Exclusion criteria: (1) patients with hematologic malignancies, including leukaemia, lymphoma and myeloma; (2) patients with severe comorbidities, acute infections or pregnancy; (3) patients lacking data on covariates; and (4) patients without survival data available. Informed consent was obtained from all patients. The study was approved by the institutional review boards of the participating hospitals and conducted in accordance with the Declaration of Helsinki.

### 2.2 Data Collection

Demographic information (e.g., age and gender), clinical characteristics (e.g., tumour type, stage and BMI) and comprehensive laboratory data were extracted from the patients' electronic medical records at baseline. Laboratory parameters included serum Cr, C‐reactive protein (CRP), albumin, haemoglobin (Hb), white blood cell count (WBC), red blood cell count (RBC), platelet count (PLT), neutrophils, lymphocytes, total protein, high‐density lipoprotein (HDL), low‐density lipoprotein (LDL), total cholesterol (TC), triglycerides (TG) and among others.

### 2.3 Biomarker Calculations

Derived inflammatory and nutritional indices were calculated based on baseline laboratory parameters, including CRP, albumin, neutrophils, lymphocytes, platelets and total protein. These indices included the C‐reactive protein‐to‐albumin ratio (CAR), neutrophil‐to‐lymphocyte ratio (NLR), lymphocyte‐to‐CRP ratio (LCR), prognostic nutritional index (PNI), platelet‐to‐lymphocyte ratio (PLR), systemic immune‐inflammation index (SII) and modified Glasgow prognostic score (mGPS). The formulas and definitions of these indices are provided in Table S1. The Cr‐to‐CRP‐to‐Albumin Ratio (CCAR) was subsequently developed as a composite index that integrates Cr, CRP and albumin using a random forest model. CCAR values were dichotomized based on the optimal cutoff (0.56) derived from maximally selected rank statistics. Patients were stratified into low (CCAR ≤ 0.56) and high (CCAR > 0.56) risk groups for survival analysis.

### 2.4 Development of the Web‐Based CCAR Calculator

A web‐based CCAR calculator was developed to facilitate the clinical application of the CCAR index for survival prediction in cancer cachexia patients. The development process involved the following steps. 1. Model training and scaling: A random forest regression model was trained on data from the development cohort, using standardized inputs of CAR (CRP/Albumin) and serum creatinine (Cr). The feature standardization was performed using the StandardScaler function from scikit‐learn, transforming each input variable to have a mean of zero and a standard deviation of one based on the training data. The same scaler was applied during real‐time predictions by the web‐based calculator, ensuring consistency between model training and deployment. The final trained model and scaler were serialized and saved using the joblib library. 2. Backend implementation: A backend server was built using the Flask framework (Python 3.11) to handle the processing of patient data. The backend accepts patient inputs (CRP, albumin and Cr) via a POST request from the frontend. The server calculates the CAR value, standardizes the inputs using the saved scaler, and predicts the CCAR value using the trained random forest model. The backend server is hosted on the PythonAnywhere platform (https://heyangzhang.pythonanywhere.com/). 3. Frontend interface: The user interface was developed using HTML5, CSS3 and JavaScript, providing input fields for CRP, albumin and creatinine, along with a ‘Calculate’ button. Upon user submission, the frontend uses JavaScript to read the input data and sends it as a JSON request to the backend server. The backend returns the predicted CCAR value and the corresponding risk classification (low‐risk: CCAR ≤ 0.56; high‐risk: CCAR > 0.56), which is then displayed dynamically on the frontend. 4. Visualization of survival risk: To enhance clinical interpretability, a colour‐coded survival heatmap was integrated, illustrating the relationship between CCAR levels and estimated survival probabilities at 1, 3 and 5 years. Higher CCAR values corresponded with progressively lower survival probabilities, and the heatmap visually reflected the patient's risk stratification.

### 2.5 Statistical Analysis

Continuous variables were expressed as median (IQR) for non‐normally distributed data or mean ± standard deviation (SD) for normally distributed data. Differences between groups were assessed using the Mann–Whitney *U* test or Student's *t*‐test. Categorical variables were presented as percentages, and comparisons between groups were made using the chi‐square test. Survival analysis was performed using Kaplan–Meier curves, with differences between survival groups evaluated by the log‐rank test. To evaluate the prognostic significance of CCAR, we performed univariate and multivariate Cox proportional hazards regression analyses. Multivariate models adjusted for potential confounders, including age, gender, BMI, TNM stage, comorbidities (diabetes, hypertension, etc.) and treatment factors. The predictive performance of CCAR was compared with traditional markers like TNM stage and BMI using the C‐index, net reclassification index (NRI) and integrated discrimination improvement (IDI). The optimal cutoff for CCAR was determined using the maximally selected rank statistics method.

## 3 Result

### 3.1 Prognostic Relevance and Limitations of Creatinine as a Standalone Biomarker

Baseline characteristics of the entire cohort (*n* = 1367) are summarized in Table S2. The median age was 59 years (IQR: 52–67), and the majority were male (62.5%). Lung (27.3%), gastric (23.8%) and colorectal cancers (19.5%) were the most common tumor types. Nearly half (48.0%) of the patients were diagnosed at TNM stage IV, and approximately 23.0% were underweight based on BMI classification. Laboratory indicators and inflammation/nutrition‐related indices such as CRP, albumin, NLR, CAR and SII showed substantial inter‐patient variation.

**FIGURE 1:**
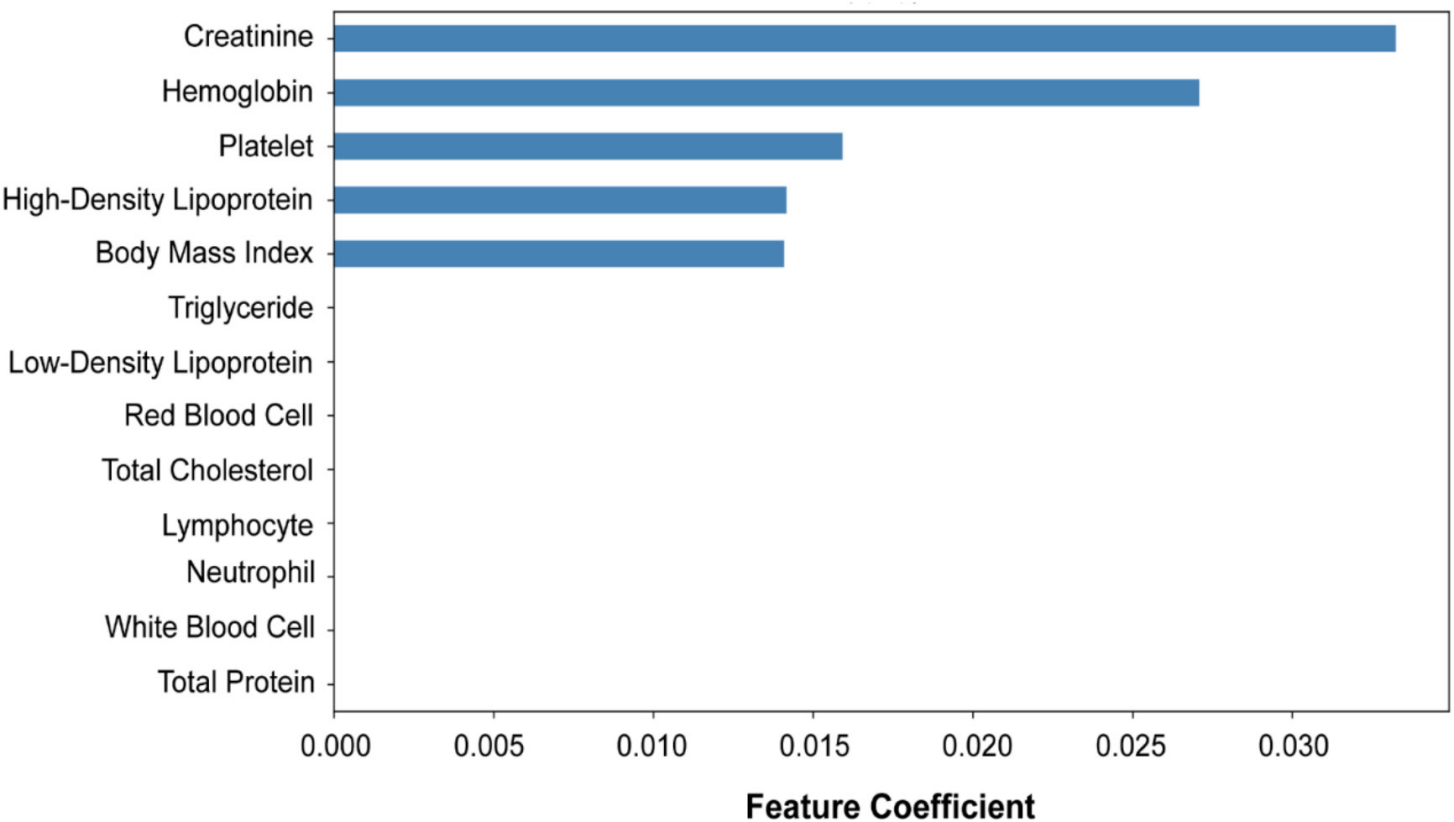
Feature importance ranked by LASSO regression in cancer cachexia patients.

To explore potential prognostic markers, we conducted LASSO regression incorporating multiple biochemical variables. Among all features, Cr demonstrated the highest predictive importance (Figure 1), suggesting its potential association with cachexia‐related survival. Patients were stratified into high and low Cr groups based on clinical thresholds (≥ 106 μmol/L for males, ≥ 97 μmol/L for females), and Kaplan–Meier (KM) analysis was performed. Elevated Cr levels were significantly associated with worse overall survival in the full cohort (*p* = 0.0012). This association remained notable in selected subgroups, including TNM stage IV (*p* = 0.074), underweight (*p* = 0.0095) and normal‐weight (*p* = 0.00052) patients, while no significant differences were observed in stages I–III or overweight groups (Figure S1). In cancer‐type–specific stratified analysis, only lung cancer (*p* = 0.0015) showed a significant Cr‐related survival difference; other tumour types did not exhibit clear prognostic separation (Figure S2). These results indicate that although Cr may reflect underlying metabolic alterations in cachexia, its prognostic value is limited and inconsistent across cancer types and clinical subgroups.

### 3.2 Cr–CAR Composite Index (CCAR) Outperforms Traditional Inflammation‐ and Nutrition‐Based Indices

Next, the entire cohort of 1367 cancer cachexia patients was randomly divided into an internal training set (*n* = 959) and an internal validation set (*n* = 408) using a 7:3 ratio. An independent external validation cohort (*n* = 284) was also included for further evaluation (Figure S3). Baseline demographic and clinicopathological characteristics across the three cohorts were generally comparable, as summarized in Table S3. The median follow‐up durations from the time of cachexia diagnosis were 20.3 months (IQR: 10.99–27.33) in the training cohort, 16.7 months (IQR: 8.36–30.17) in the internal validation cohort, and 16.7 months (IQR: 7.97–28.82) in the external validation cohort. In addition, 98 patients (68 in the training cohort, 25 in the internal validation cohort, and 5 in the external validation cohort) had follow‐up beyond 5 years, providing direct observations for long‐term survival estimates. To identify the most reliable prognostic indicator among existing inflammation‐ and nutrition‐related biomarkers, we compared the predictive ability of 10 commonly used indices, including CAR, NLR, LCR, PNI, SII, mGPS, and etc. Across internal training, internal validation and external validation cohorts, CAR consistently showed the highest C‐index (0.627, 0.656 and 0.660, respectively) (Table S4). Time‐dependent ROC analysis further confirmed that CAR maintained the highest AUC values throughout the follow‐up period in all three cohorts (Figure S4), suggesting its superior and stable predictive ability in cancer cachexia patients.

Given the limited standalone prognostic value of Cr and the robust predictive performance of CAR across all cohorts, we sought to integrate these two indicators using a machine learning approach. Specifically, we constructed a composite index termed Cr–CAR composite index (CCAR), based on Cr, CRP and albumin, via a random forest model. As shown in Table 1, CCAR exhibited a markedly superior prognostic ability compared with either CAR or Cr alone, with consistently higher C‐index values in the internal training (0.777), internal validation (0.789) and external validation cohorts (0.765). Time‐dependent AUC curves further confirmed the improved discrimination of CCAR across all three cohorts (Figure S5). When compared against 11 other established inflammation and nutrition related indices (e.g., LCR, NLR, mGPS, SII, PNI), CCAR consistently ranked first in both C‐index (Table S5) and time‐dependent AUC performance (Figure S6), demonstrating its robust and stable prognostic value for cancer cachexia patients.

**TABLE 1:** C‐index and 95% confidence intervals for prognostic models in different cohorts.

Internal training cohort				Internal validation cohort				External validation cohort			
	C‐index	Lower 95	Upper 95		C‐index	Lower 95	Upper 95		C‐index	Lower 95	Upper 95
CCAR	0.777	0.757	0.798	CCAR	0.789	0.757	0.820	CCAR	0.765	0.728	0.803
CAR	0.627	0.599	0.654	CAR	0.649	0.607	0.692	CAR	0.660	0.613	0.708
Cr	0.511	0.482	0.541	Cr	0.492	0.447	0.538	Cr	0.518	0.467	0.569

### 3.3 Validation of CCAR as a Prognostic Biomarker for Cancer Cachexia

**FIGURE 2:**
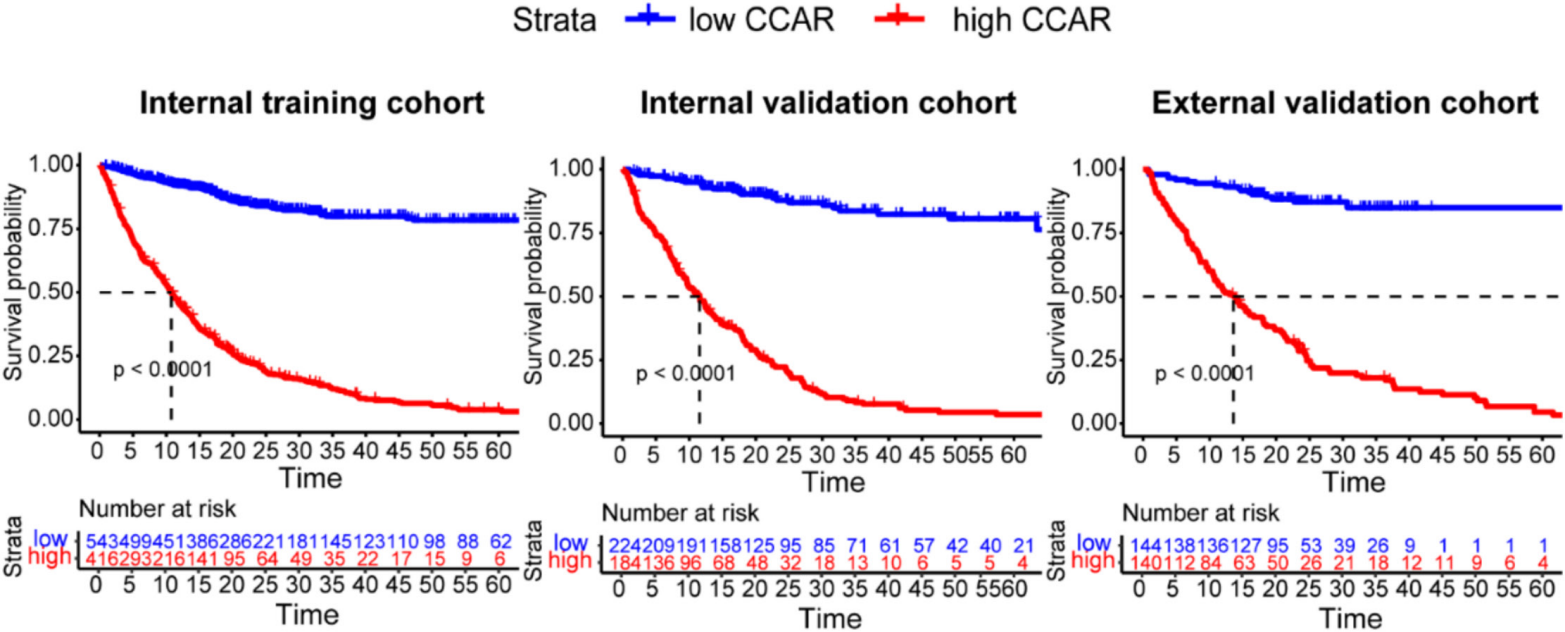
Kaplan–Meier survival curves for low and high CCAR groups in different Cohorts.

To assess the prognostic value of the CCAR index, we determined the optimal cutoff using the maximally selected rank statistics method, identifying a cutoff point at 0.56 (Figure S7). Kaplan–Meier survival analysis demonstrated that higher CCAR levels were significantly associated with poorer overall survival across the entire cohort, as well as in the internal training and external validation cohorts (Figure 2). The survival differences were evident in both the internal training cohort (*p* < 0.0001) and internal validation cohort (*p* < 0.0001), with similar findings in the external validation cohort (*p* < 0.0001). Furthermore, stratified survival analysis revealed that CCAR effectively discriminated survival outcomes across various TNM stages, BMI categories and cancer types, with significant survival differences particularly observed in TNM stages III/IV and lung cancer patients (Figure S8 and Figure S9). These findings underscore the robustness of CCAR as a predictive biomarker for cancer cachexia in diverse clinical and cancer subgroups.

### 3.4 CCAR as an Independent Prognostic Biomarker in Cancer Cachexia

To assess the prognostic value of the CCAR index, we examined the relationship between CCAR and survival outcomes. Figure S10 shows that higher CCAR values are consistently linked to worse survival outcomes across all cohorts (internal training, internal validation and external validation) with HRs increasing as CCAR rises (*p* < 0.001). Figure S11 illustrates the results from two models (a and b), both of which confirm that increasing CCAR values are associated with higher HRs.

Table 2 presents the univariate and multivariate survival analysis results for various clinicopathological variables across the training, validation and external cohorts. In the univariate analysis, age ≥ 65 years was significantly associated with worse survival in the training (HR = 1.42, *p* < 0.001) and external cohorts (HR = 1.54, *p* = 0.009), but this association was no longer significant in the multivariate analysis, suggesting that age's effect is attenuated when adjusted for other variables. Gender also showed a protective effect for females in the univariate analysis (HR = 0.79, *p* = 0.016), but this effect diminished in the multivariate analysis (HR = 1.01, *p* = 0.933), highlighting the impact of other factors on survival. Tumour stage remained a strong prognostic factor, with stage IV patients showing significantly worse survival outcomes in both univariate (HR = 7.36, *p* < 0.001) and multivariate models (HR = 2.83, *p* = 0.002). CCAR, a composite index, was a significant and consistent predictor of survival across all cohorts, with high CCAR values associated with a markedly higher hazard of death (HR = 3.31, *p* < 0.001 in the training cohort, HR = 3.51, *p* < 0.001 in the validation cohort, and HR = 3.21, *p* < 0.001 in the external cohort), confirming its robust predictive value even after adjusting for various clinical factors.

**TABLE 2:** Univariate and multivariate survival analysis of clinicopathological characteristics.

Variable	Level	Model	Training cohort HR (95% CI)	*p*	Validation cohort HR (95% CI)	*p*	External cohort HR (95% CI)	*p*
Age ≥ 65		Model a	1.42 (1.17, 1.72)	< 0.001	1.02 (0.76, 1.36)	0.897	1.54 (1.11, 2.14)	0.009
		Model b	1.01 (1.00, 1.02)	0.084	1.00 (0.99, 1.02)	0.522	1.01 (0.99, 1.03)	0.306
Gender (female)		Model a	0.79 (0.65, 0.96)	0.016	0.74 (0.55, 0.99)	0.042	0.67 (0.47, 0.95)	0.024
		Model b	1.01 (0.80, 1.27)	0.933	0.96 (0.65, 1.43)	0.853	0.68 (0.42, 1.10)	0.112
Family history (yes)		Model a	0.76 (0.57, 1.01)	0.062	0.84 (0.58, 1.21)	0.352	1.41 (0.98, 2.03)	0.064
		Model b	1.09 (0.81, 1.46)	0.572	0.93 (0.62, 1.37)	0.701	1.26 (0.85, 1.87)	0.254
Smoking (yes)		Model a	1.20 (1.02, 1.42)	0.029	1.48 (1.14, 1.91)	0.081	1.31 (0.94, 1.82)	0.117
		Model b	1.18 (0.94, 1.48)	0.165	1.13 (0.76, 1.67)	0.552	0.72 (0.46, 1.13)	0.154
Drinking (yes)		Model a	0.94 (0.76, 1.17)	0.606	1.18 (0.86, 1.63)	0.288	1.21 (0.84, 1.72)	0.306
		Model b	0.77 (0.60, 0.99)	0.039	1.09 (0.75, 1.59)	0.636	1.11 (0.73, 1.70)	0.623
Tumour stage	I		Ref.		Ref.		Ref.	
	II	Model a	1.72 (0.86,3.42)	0.125	1.98 (0.74,5.30)	0.175	1.24 (0.15, 10.70)	0.839
	II	Model b	1.30 (0.65, 2.61)	0.456	1.89 (0.69, 5.16)	0.217	1.23 (0.14, 10.97)	0.847
	III	Model a	2.77 (1.45, 5.32)	0.003	4.09 (1.64, 10.20)	0.003	1.52 (0.20, 11.27)	0.683
	III	Model b	1.86 (0.97, 3.63)	0.062	1.90 (0.74, 4.88)	0.180	1.46 (0.19, 11.37)	0.719
	IV	Model a	7.36 (3.90, 13.89)	< 0.001	7.90 (3.22, 19.41)	< 0.001	4.67 (0.65, 33.50)	0.125
	IV	Model b	2.83 (1.47, 5.43)	0.002	2.76 (1.09, 6.99)	0.033	2.98 (0.40, 22.40)	0.290
BMI	<18.5		Ref.		Ref.		Ref.	
	18.5–23.9	Model a	0.86 (0.69, 1.08)	0.189	0.76 (0.53, 1.08)	0.130	0.72 (0.51, 1.01)	0.060
	18.5–23.9	Model b	0.85 (0.68, 1.07)	0.156	0.93 (0.57, 1.58)	0.708	0.93 (0.64, 1.36)	0.718
	23.9–28.0	Model a	0.85 (0.63, 1.14)	0.283	0.59 (0.37, 0.93)	0.023	0.76 (0.37, 1.53)	0.438
	23.9–28.0	Model b	0.80 (0.58, 1.09)	0.161	0.94 (0.56, 1.58)	0.827	0.87 (0.41, 1.81)	0.702
	>28	Model a	0.17 (0.04, 0.67)	0.012	0.39 (0.12, 1.26)	0.115	NA	NA
	>28	Model b	0.40 (0.10, 1.64)	0.204	0.54 (0.16, 1.81)	0.319	NA	NA
Chemotherapy (yes)		Model a	0.84 (0.69, 1.01)	0.063	0.99 (0.74, 1.32)	0.941	0.63 (0.45, 0.89)	0.008
		Model b	0.79 (0.64, 0.98)	0.034	0.62 (0.43, 0.90)	0.011	0.77 (0.53, 1.14)	0.193
Radiotherapy (yes)		Model a	0.84 (0.57, 1.23)	0.368	0.82 (0.47, 1.44)	0.487	2.16 (0.95, 4.90)	0.066
		Model b	0.99 (0.66, 1.49)	0.958	0.62 (0.32, 1.19)	0.152	1.59 (0.66, 3.83)	0.298
Diabetes (yes)		Model a	1.31 (0.99, 1.73)	0.063	1.29 (0.84, 2.00)	0.251	1.21 (0.72, 2.04)	0.476
		Model b	1.14 (0.85, 1.52)	0.389	1.11 (0.67, 1.84)	0.693	0.98 (0.55, 1.76)	0.957
Hypertension (yes)		Model a	1.23 (0.97, 1.56)	0.094	1.21 (0.87, 1.68)	0.264	1.03 (0.71, 1.50)	0.883
		Model b	1.03 (0.79, 1.33)	0.831	0.90 (0.61, 1.32)	0.577	1.02 (0.65, 1.58)	0.939
Coronary disease (yes)		Model a	1.59 (1.09, 2.34)	0.017	0.88 (0.42, 1.84)	0.732	0.57 (0.80, 4.1)	0.579
		Model b	1.25 (0.83, 1.90)	0.273	0.87 (0.37, 2.07)	0.753	1.81 (0.24, 13.72)	0.568
CCAR (> 0.56)		Model a	3.31 (2.94, 3.72)	< 0.001	3.51 (2.92, 4.22)	< 0.001	3.21 (2.55, 4.03)	< 0.001
		Model b	2.97 (2.63, 3.36)	< 0.001	3.63 (2.94, 4.47)	< 0.001	3.00 (2.33, 3.87)	< 0.001

*Note:* Model a: No adjusted. Model b: Adjusted for age, gender, BMI, TNM stage, surgery, coronary heart disease, drinking, gender, family history, smoking, chemotherapy, radiotherapy, diabetes and hypertension.

### 3.5 Enhanced Predictive Capability of the CCAR Index Compared With Traditional Biomarkers

To further assess the predictive capability of CCAR compared with traditional biomarkers, we conducted an analysis of the Net Reclassification Index (NRI) and Integrated Discrimination Improvement (IDI) for CCAR, TNM stage and BMI (Table S6). Adding CCAR to the TNM stage–based model significantly improved risk prediction, with NRI = 0.490 (*p* < 0.001) and IDI = 0.269 (*p* < 0.001). Similarly, the inclusion of CCAR into the BMI‐based model resulted in substantial gains, with NRI = 1.171 (*p* < 0.001) and IDI = 0.410 (*p* < 0.001). These results demonstrate that CCAR provides incremental prognostic value when integrated with conventional clinical indicators. Notably, since CCAR itself is a composite index incorporating creatinine, CRP and albumin, it can also be applied independently as a simple and robust prognostic tool in clinical settings. Additionally, we assessed the distribution of CCAR across various tumour stages and BMI categories (Figure S12). Significant variations in CCAR levels were observed across tumour stages (*p* < 2.2e‐16), with higher CCAR values correlating with advanced stages (III and IV) and lower values seen in earlier stages (I and II). Similarly, CCAR levels significantly differed across BMI categories (*p* = 0.02), with overweight and obese patients having lower CCAR levels, while underweight and normal‐weight patients exhibited higher levels. In Figure S13, we explored the distribution of CCAR across various tumour types, revealing that certain cancers, such as lung and colorectal cancer, had significantly higher CCAR levels compared with others, reinforcing the association between CCAR and cachexia severity. These findings underscore the sensitivity of CCAR to clinical variations and its ability to discriminate between different levels of cachexia severity across both tumour stages and BMI categories.

### 3.6 Development and Application of the CCAR‐Based Web Tool for Survival Prediction

**FIGURE 3:**
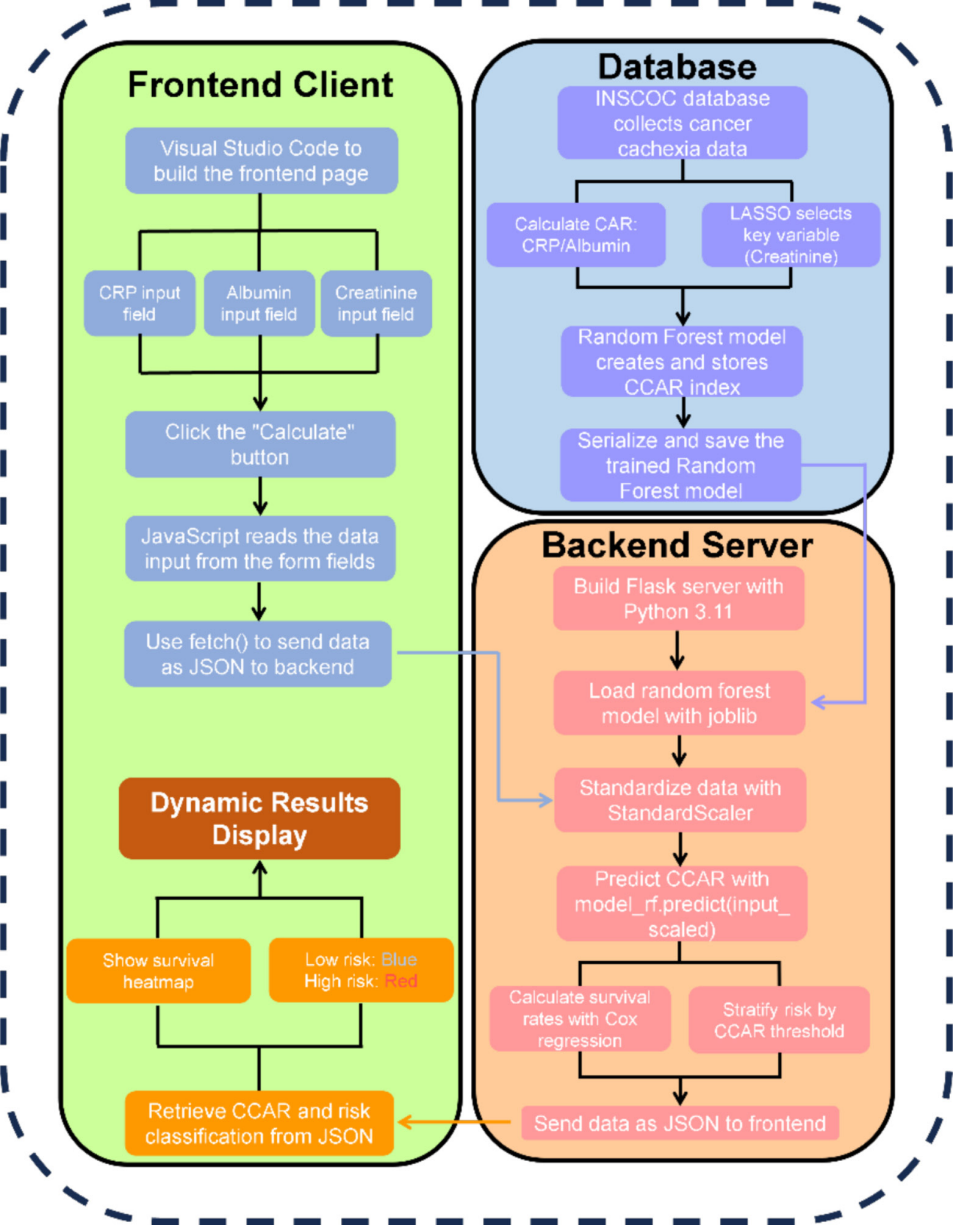
Schematic of the web‐based CCAR calculator workflow.

**FIGURE 4:**
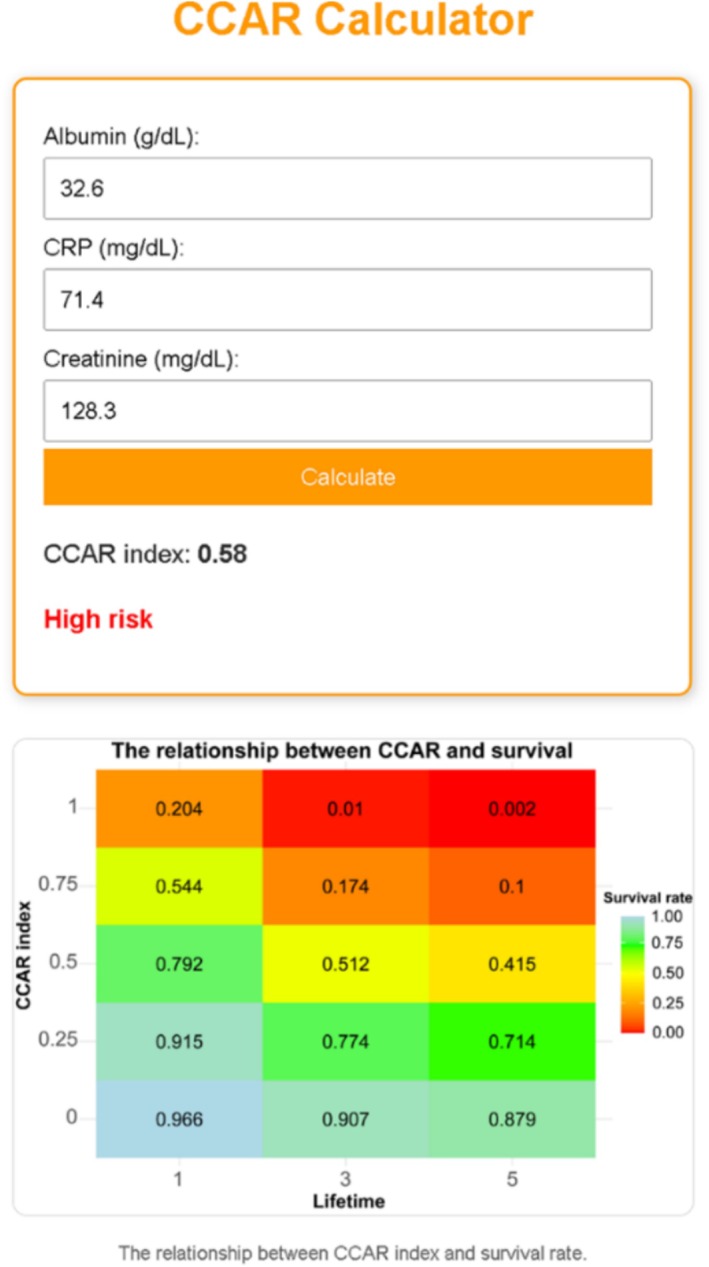
CCAR calculator interface and heatmap visualization for survival prediction in cancer cachexia.

To facilitate the clinical application of the CCAR index in assessing cancer cachexia prognosis, we developed a user‐friendly web‐based calculator that utilizes a random forest model for risk prediction (Figure 3). Figure 4 illustrates the functionality of the CCAR calculator, where clinicians input the patient's albumin (g/dL), CRP (mg/dL) and Cr (mg/dL) levels. Upon clicking the ‘Calculate’ button, the system processes these inputs using the trained random forest model to generate the CCAR index and associated risk level. The patient's 1‐, 3‐ and 5‐year survival rates can be assessed based on a colour‐coded heatmap. This web‐based tool integrates complex machine learning predictions into an accessible format, offering clinicians an efficient and reliable method to assess cancer cachexia risk and forecast patient survival.

## 4 Discussion

Cancer cachexia is a complex multifactorial syndrome characterized by involuntary weight loss, muscle wasting and metabolic dysfunction, leading to poor patient outcomes [2]. The prognostic assessment of cancer cachexia has remained challenging due to the lack of universally accepted biomarkers that can accurately predict disease progression and survival. In this study, we developed a novel predictive model, CCAR, by integrating three widely used clinical biomarkers—Cr, CRP and albumin—using a machine learning approach (random forest model). Multicenter validation confirmed the clinical superiority of CCAR.

The paradoxical prognostic value of serum Cr in cancer cachexia reflects its complex biological underpinnings. As a byproduct of phosphocreatine degradation in muscle tissue, Cr levels typically correlate positively with muscle mass [12]. In healthy individuals, Cr is excreted at a relatively constant rate via the kidneys, rendering it a reliable surrogate marker for skeletal muscle mass and metabolic activity [13]. Several studies have reported that higher Cr concentrations are associated with greater muscle mass and enhanced muscle function, particularly in stable or healthy populations [14]. However, in the context of cancer cachexia, Cr levels may fluctuate due to the intricate interplay between muscle wasting, metabolic derangement and systemic inflammation [15]. Under inflammatory stimuli, pro‐inflammatory cytokines such as TNF‐α and IL‐6 are markedly elevated, which in turn activate proteolytic pathways in muscle cells, including the ubiquitin–proteasome system and the [16, 17, 18, 19]. These catabolic cascades accelerate muscle protein breakdown and promote rapid conversion of phosphocreatine into Cr, potentially leading to transient elevations in serum Cr [20, 21]. Additionally, cachexia is often accompanied by subclinical renal impairment, which reduces Cr clearance and further contributes to elevated serum concentrations [22]. Therefore, increased Cr levels in cachectic patients may not simply indicate preserved muscle mass, but rather a combined consequence of acute muscle proteolysis, inflammatory imbalance and impaired metabolic clearance. This mechanistic insight aligns with our observation that patients with elevated Cr levels exhibited significantly worse overall survival, particularly in subgroups with low BMI or advanced TNM staging. The incorporation of Cr into the CCAR index was based on its unique metabolic positioning within the cachexia pathophysiology, thereby enhancing the index's ability to capture the inflammatory, nutritional and metabolic dimensions of disease progression in a mechanistically relevant manner.

Clinically, Cr has been proposed as a potential biomarker for cancer cachexia and muscle wasting in various malignancies. A large dual‐cohort study from the PREVEND and NHANES datasets demonstrated a positive correlation between serum Cr and muscle mass, with stronger predictive value after adjusting for cystatin C. Notably, elevated Cr levels were associated with a lower risk of mortality, suggesting its potential utility as a muscle mass surrogate rather than a renal function marker [23]. Another large‐scale study involving 3060 cancer patients reported that the creatinine‐to‐cystatin C ratio at diagnosis significantly predicted both 6‐month and 1‐year mortality. Higher ratios were consistently associated with reduced mortality risk and shorter ICU and hospital stays, even after adjusting for confounders [24]. These findings imply that Cr may serve as a prognostic indicator of muscle reserve and clinical outcomes. However, our findings suggest that the prognostic role of Cr in cancer cachexia is more nuanced. Although Cr is conventionally regarded as a positive indicator of muscle mass, in the cachectic setting, its elevation may more accurately reflect acute catabolism, systemic inflammation and renal dysfunction. In our study, patients in the high Cr group (≥ 106 μmol/L for males, ≥ 97 μmol/L for females) exhibited significantly worse survival (*p* = 0.0012), with particularly pronounced predictive power among patients with TNM stage IV (*p* = 0.074) and low BMI (*p* = 0.0095). Notably, the prognostic utility of Cr disappeared in overweight patients (*p* = 0.26), likely due to the masking effect of adipose tissue on muscle loss—a phenomenon that supports the ‘obesity paradox’ in cachexia. Furthermore, the stability of Cr as a stand‐alone prognostic biomarker is challenged by its susceptibility to multiple physiological influences, including skeletal muscle mass, renal function and systemic inflammation.

The rationale for combining Cr with CAR lies in their complementary pathophysiological dimensions. CAR, a well‐established inflammation‐nutrition index, reflects systemic inflammatory burden (via CRP) and hepatic synthetic function, while Cr quantifies muscle catabolism, a hallmark of cachexia progression [12, 25, 26, 27]. CAR is sensitive to acute inflammatory events, whereas Cr reflects chronic muscle wasting [28, 29, 30, 31]. By integrating Cr and CAR (CRP/albumin) through a random forest model, the CCAR index simultaneously captures three key pathological dimensions of cachexia: inflammation, nutrition and metabolism. As shown in Table 1, CCAR demonstrated significantly superior performance across three validation cohorts (C‐index: 0.765–0.789) compared with CAR (0.627–0.660) or Cr (0.492–0.518) alone. This stability stems from its biological plausibility—Figure S12 reveals that CCAR levels increase stepwise with tumour progression (stage I → IV; *p* < 2.2e−16). Moreover, NRI/IDI analysis (Table S6) quantified CCAR's incremental value: compared with TNM staging, CCAR correctly reclassified 49% of patients (NRI = 0.490) and improved risk discrimination by 26.9% (IDI = 0.269). CCAR's predictive power surpasses traditional TNM staging, carrying critical clinical implications. In early‐stage patients, elevated CCAR (> 0.56) may indicate occult metabolic dysregulation, warranting early nutritional support, exercise rehabilitation or anti‐inflammatory therapy to halt cachexia progression. Conversely, in advanced‐stage patients, low CCAR (≤ 0.56) may identify those likely to benefit from aggressive treatment.

We also developed a web‐based calculator that integrates real‐time laboratory data with machine learning algorithms to rapidly compute CCAR values and risk stratification. Its colour‐coded visualization effectively displays survival probabilities, offering clinicians an evidence‐based, user‐friendly tool for risk stratification, personalized intervention and treatment monitoring. In addition to static risk stratification, future applications of CCAR could explore its longitudinal monitoring potential, including integration with dynamic biomarkers and imaging modalities such as CT‐derived skeletal muscle indices. Future studies should validate CCAR in larger, more diverse cohorts, explore its potential for guiding therapeutic interventions, and investigate its integration with emerging biomarkers or imaging techniques to further enhance predictive accuracy and solidify its role in cachexia management.

## 5 Limitation

This study has several limitations. First, the biological mechanisms underlying CCAR require further validation through histopathological and mechanistic studies. Second, we did not assess longitudinal changes in CCAR, which could provide insight into its dynamic prognostic value over the course of disease. Third, the majority of patients in our cohort were of Asian descent, highlighting the need for external validation in Western populations to confirm the generalizability of our findings. Finally, because only patients with an established diagnosis of cancer cachexia were included, the CCAR index should be regarded as a prognostic tool after diagnosis, and its role in predicting the initial onset of cachexia requires further study.

## 6 Conclusion

In conclusion, the CCAR index—a composite measure of Cr, C‐reactive protein and albumin—emerges as a powerful and reliable prognostic tool for cancer cachexia. It outperforms traditional inflammation‐ and nutrition‐based biomarkers in predicting survival outcomes and is readily applicable in clinical practice. The development of a web‐based CCAR calculator further facilitates its implementation, offering a practical approach for real‐time prognostic evaluation in cancer cachexia management.

## Ethics Statement

This study followed the Helsinki declaration. All participants signed an informed consent form, and this study was approved by the Institutional Review Board of each hospital (registration number: ChiCTR1800020329).

## Conflicts of Interest

The authors declare no conflicts of interest.

## Supporting information


**Figure S1:** Survival curves of different cohorts based on Cr levels.
**Figure S2:** Survival curves stratified by cancer types based on Cr levels.
**Figure S3:** Flow chart.
**Figure S4:** Comparison of AUC scores for different inflammatory and nutritional indices.
**Figure S5:** AUC comparison of CCAR with CAR and Cr.
**Figure S6:** AUC comparison of CCAR with traditional prognostic indicators.
**Figure S7:** Cutoff value of CCAR index.
**Figure S8:** Survival curves of different cohorts based on CCAR levels.
**Figure S9:** Cancer type‐specific stratified survival curves based on CCAR.
**Figure S10:** Log‐hazard ratio of CCAR for cancer cachexia prognosis.
**Figure S11:** Log‐hazard ratio of CCAR for cancer cachexia prognosis in different model.
**Figure S12:** The distribution of CCAR index in tumour stage and BMI.
**Figure S13:** The distribution of CCAR index in different tumour type.
**Table S1:** Inflammatory biomarkers evaluated in this study.
**Table S2:** Demographic and clinical characteristics of the study population.
**Table S3:** Comparison of demographic and clinicopathological characteristics across different cohorts.
**Table S4:** C‐index values of various biomarkers in different cohorts.
**Table S5:** C‐index values of CCAR, CAR, NLR, LCR, mGPS, PNI and mGNRI in different cohorts.
**Table S6:** Comparison of the prediction ability among different models through NRI and IDI.

## Data Availability

The datasets used and/or analysed during the current study are available from the corresponding author on reasonable request.
