# Supplementary material for: A Creatinine–CAR Composite Index (CCAR) Optimized by Machine Learning for Prognosis in Cancer Cachexia
Source: J Cachexia Sarcopenia Muscle. 2025 Nov 11;16(6):e70120. doi: 10.1002/jcsm.70120 (PMC12603776; doi:10.1002/jcsm.70120)
Supplement: Supplementary file 1 — Figure S1: Survival curves of different cohorts based on Cr levels. Figure S2: Survival curves stratified by cancer types based on Cr levels. Figure S3: Flow chart. Figure S4: Comparison of AUC scores for different inflammatory and nutritional indices. Figure S5: AUC comparison of CCAR with CAR and Cr. Figure S6: AUC comparison of CCAR with traditional prognostic indicators. Figure S7: Cutoff value of CCAR index. Figure S8: Survival curves of different cohorts based on CCAR levels. Figure S9: Cancer type‐specific stratified survival curves based on CCAR. Figure S10: Log‐hazard ratio of CCAR for cancer cachexia prognosis. Figure S11: Log‐hazard ratio of CCAR for cancer cachexia prognosis in different model. Figure S12: The distribution of CCAR index in tumour stage and BMI. Figure S13: The distribution of CCAR index in different tumour type. Table S1: Inflammatory biomarkers evaluated in this study. Table S2: Demographic and clinical characteristics of the study population. Table S3: Comparison of demographic and clinicopathological characteristics across different cohorts. Table S4: C‐index values of various biomarkers in different cohorts. Table S5: C‐index values of CCAR, CAR, NLR, LCR, mGPS, PNI and mGNRI in different cohorts. Table S6: Comparison of the prediction ability among different models through NRI and IDI. [file JCSM-16-e70120-s001.docx]

**Supplementary figures**

**Figure S1.** **Survival curves of different cohorts based on Cr levels.**


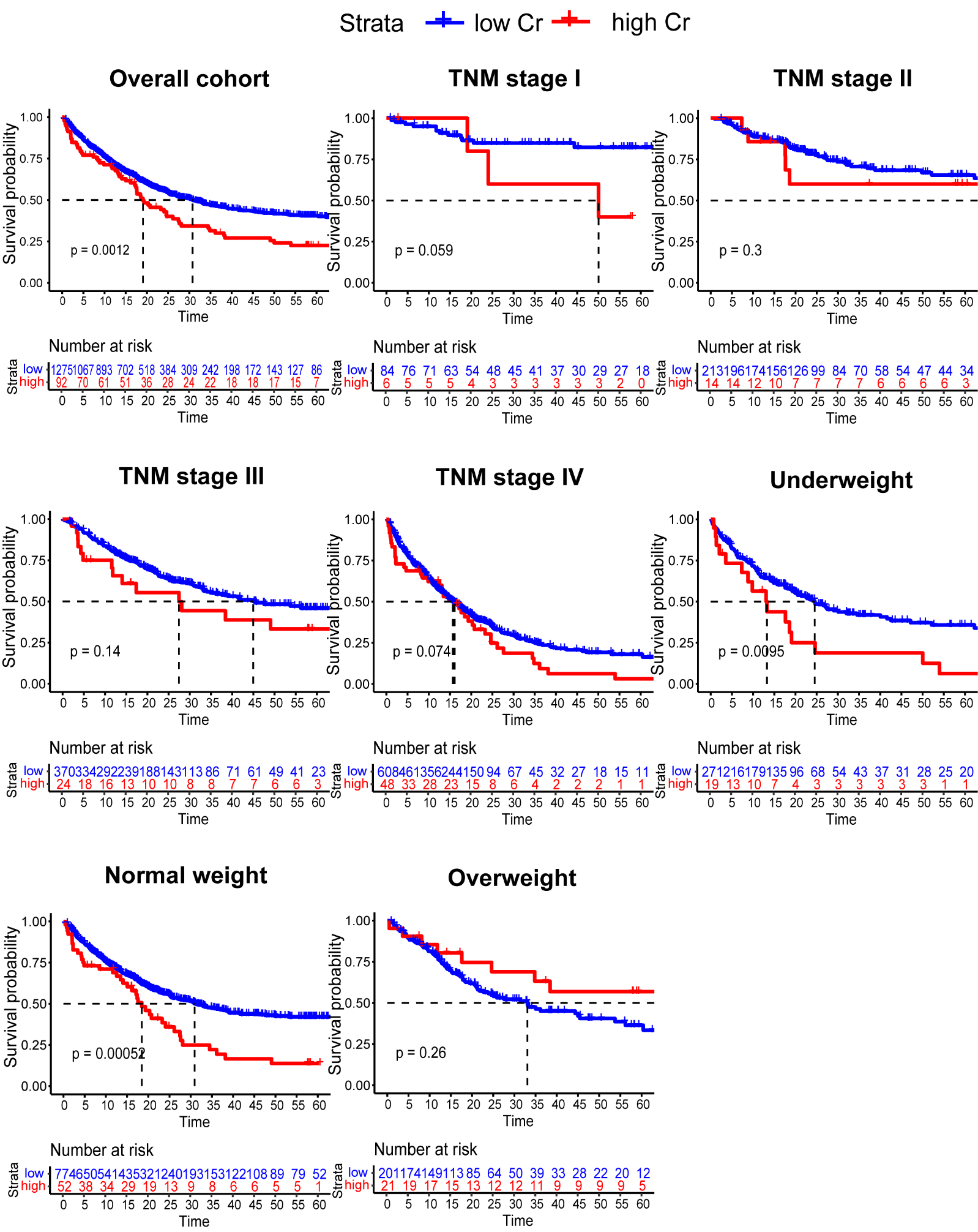


**Figure S2.** **Survival curves stratified by cancer types based on Cr levels.**


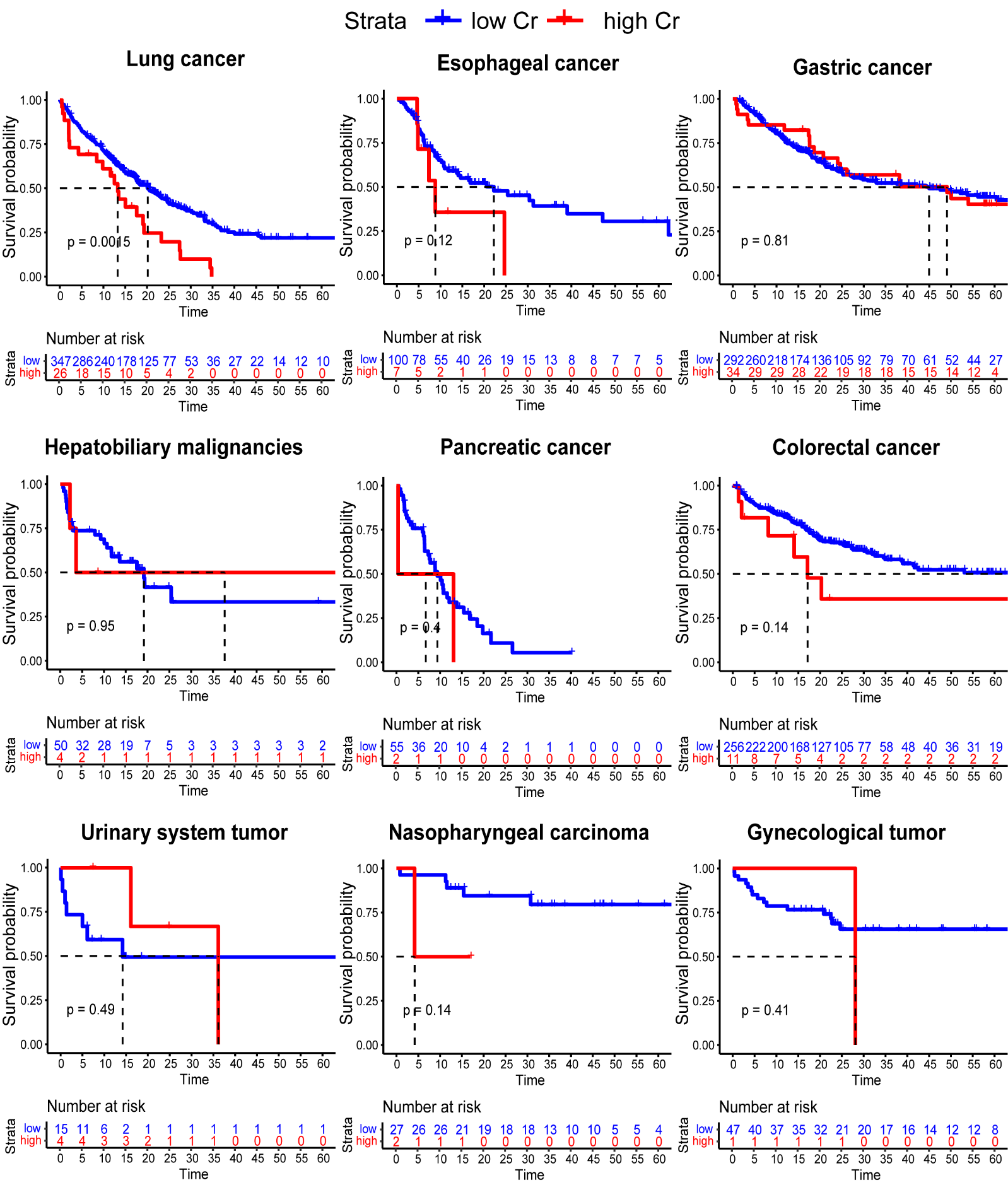


**Figure S3.** **Flow chart.**


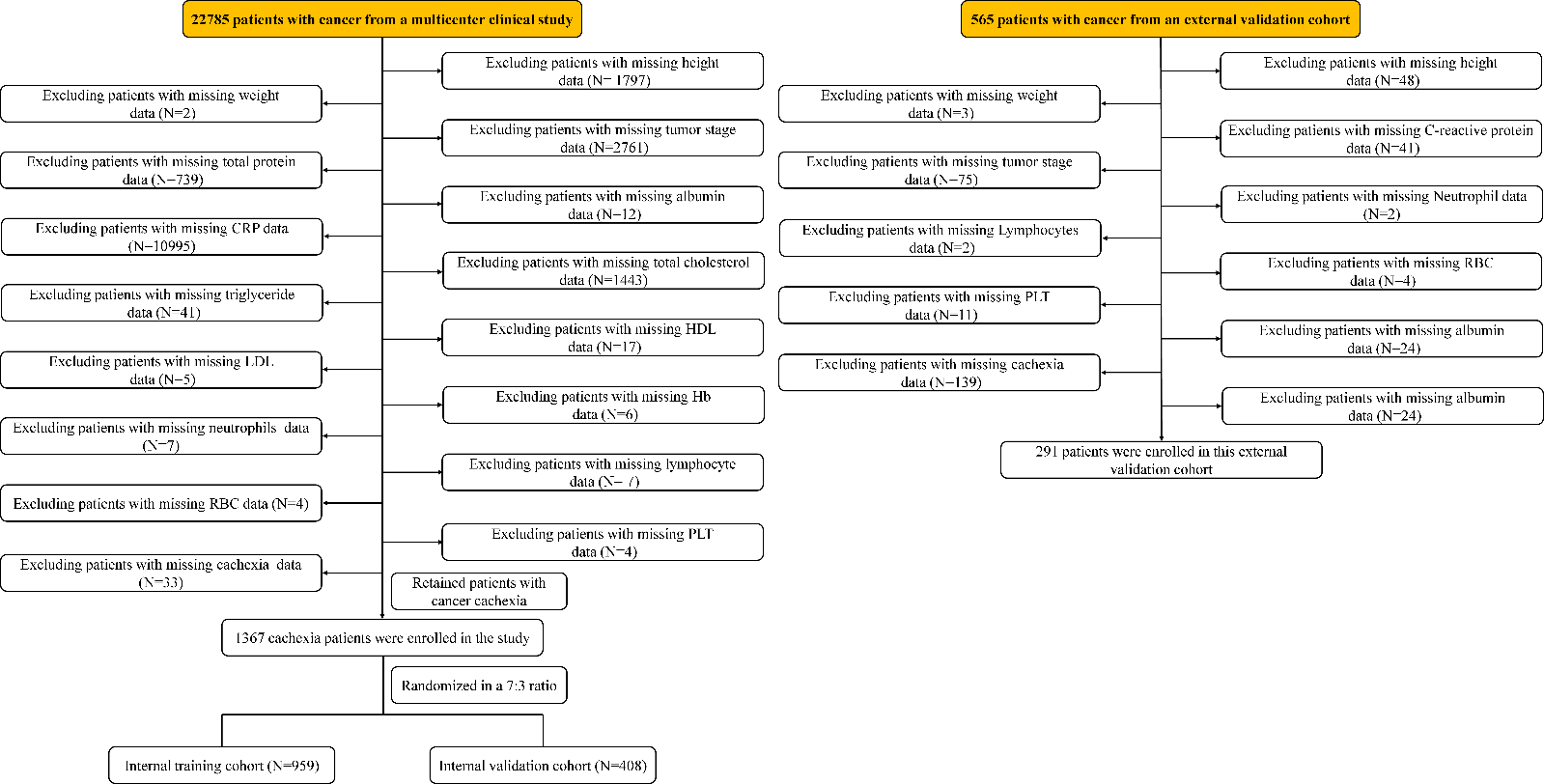


**Figure S4. Comparison of AUC scores for different inflammatory and nutritional indices.**


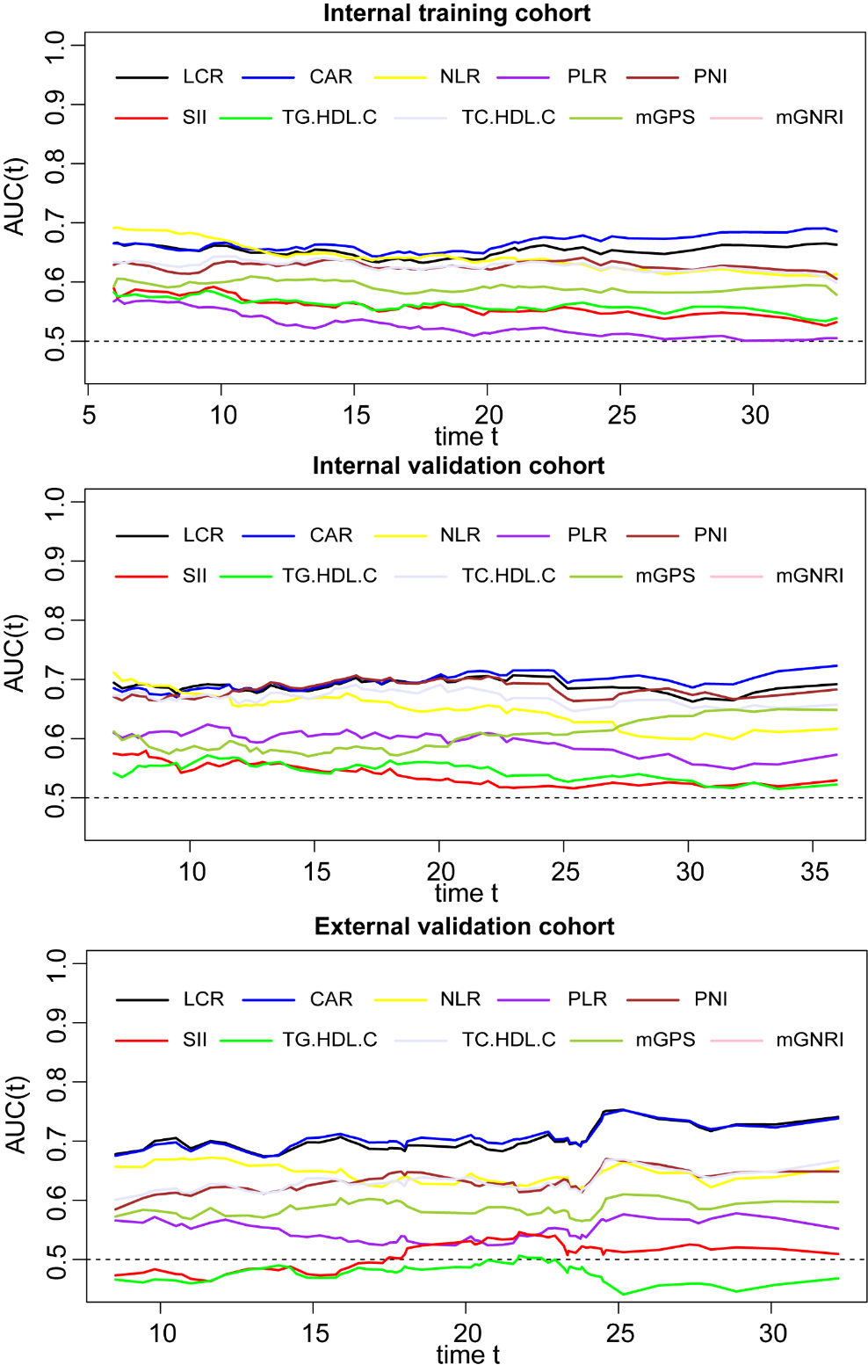


**Figure S5. AUC comparison of CCAR with CAR and Cr.**


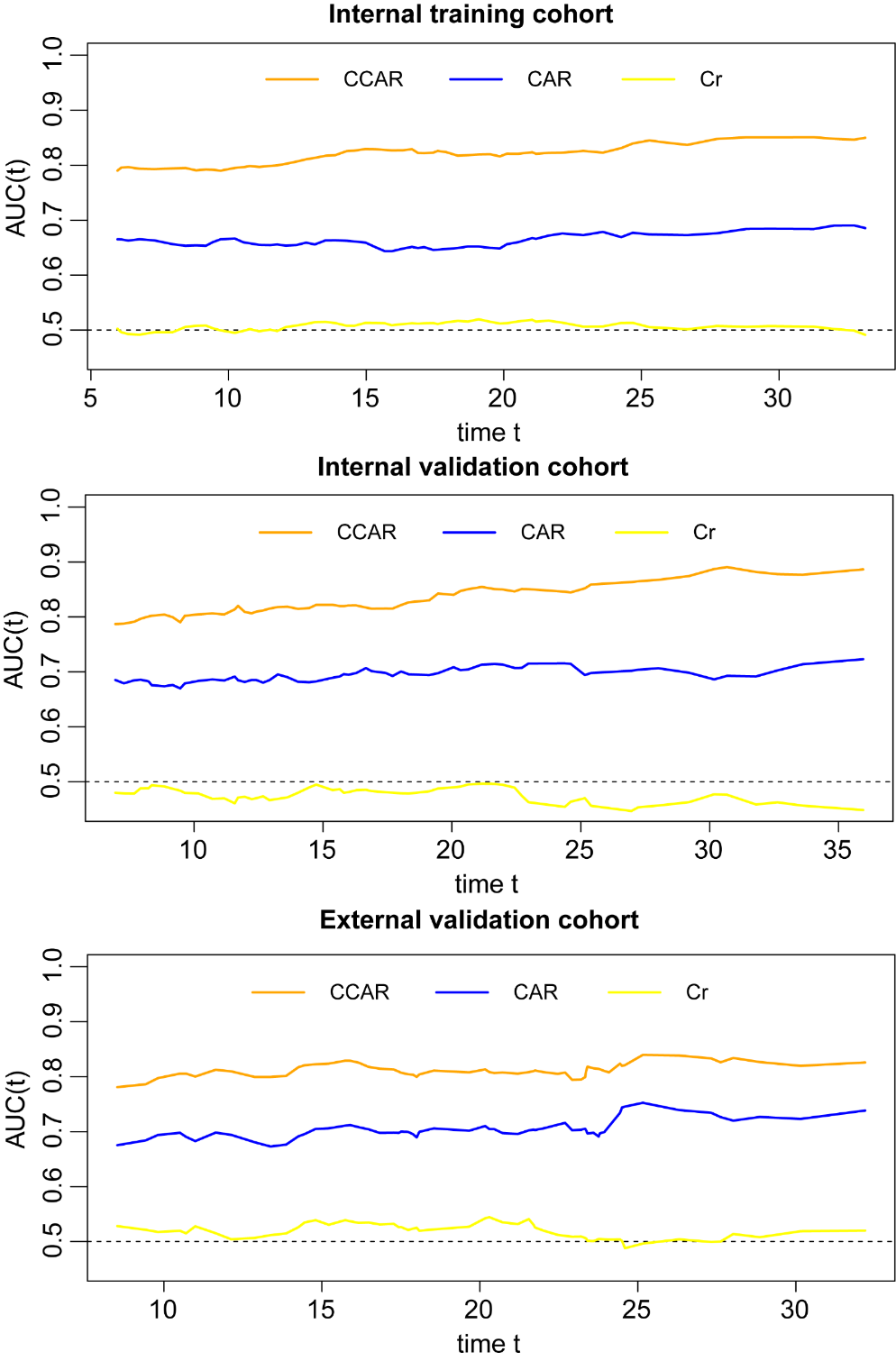


**Figure S6. AUC comparison of CCAR with traditional prognostic indicators.**


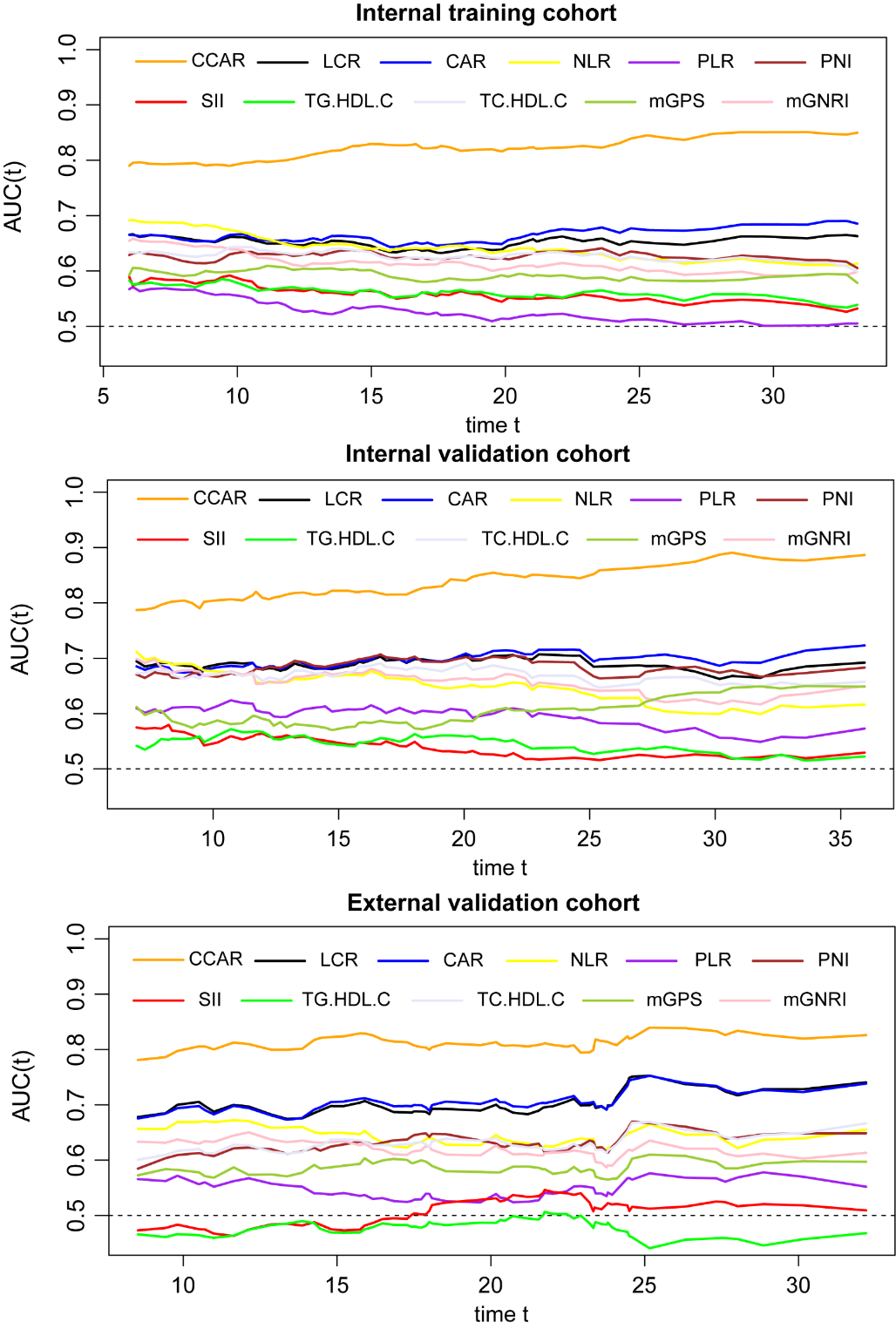


**Figure S7. Cutoff value of CCAR index.**


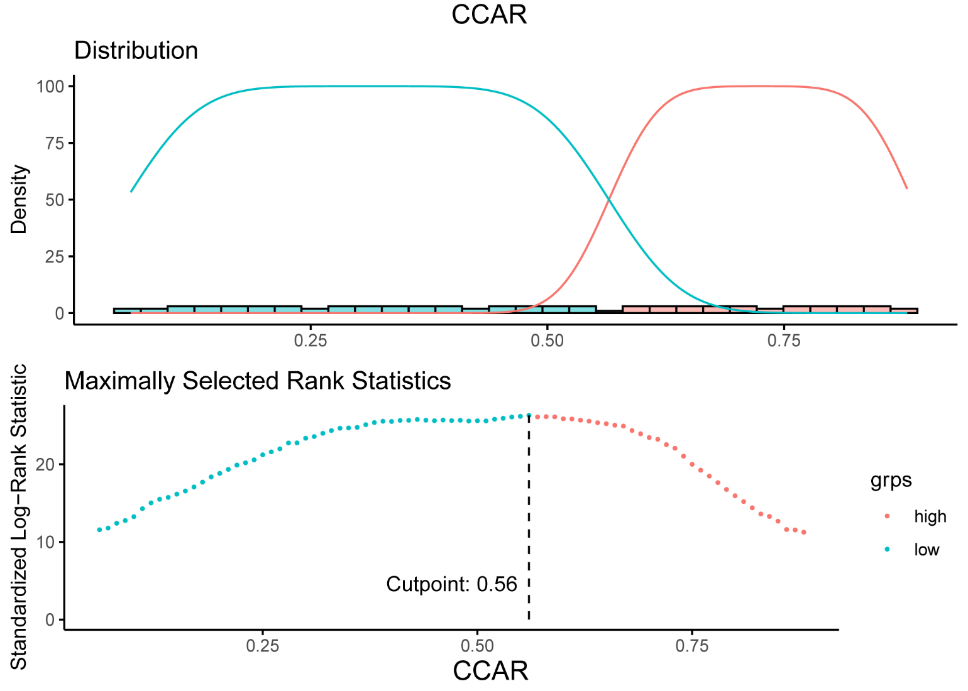


Notes: CCAR, Creatinine-C-Reactive protein-Albumn-Ratio based Combination Indicator.

**Figure S8. Survival curves of different cohorts based on CCAR levels.**


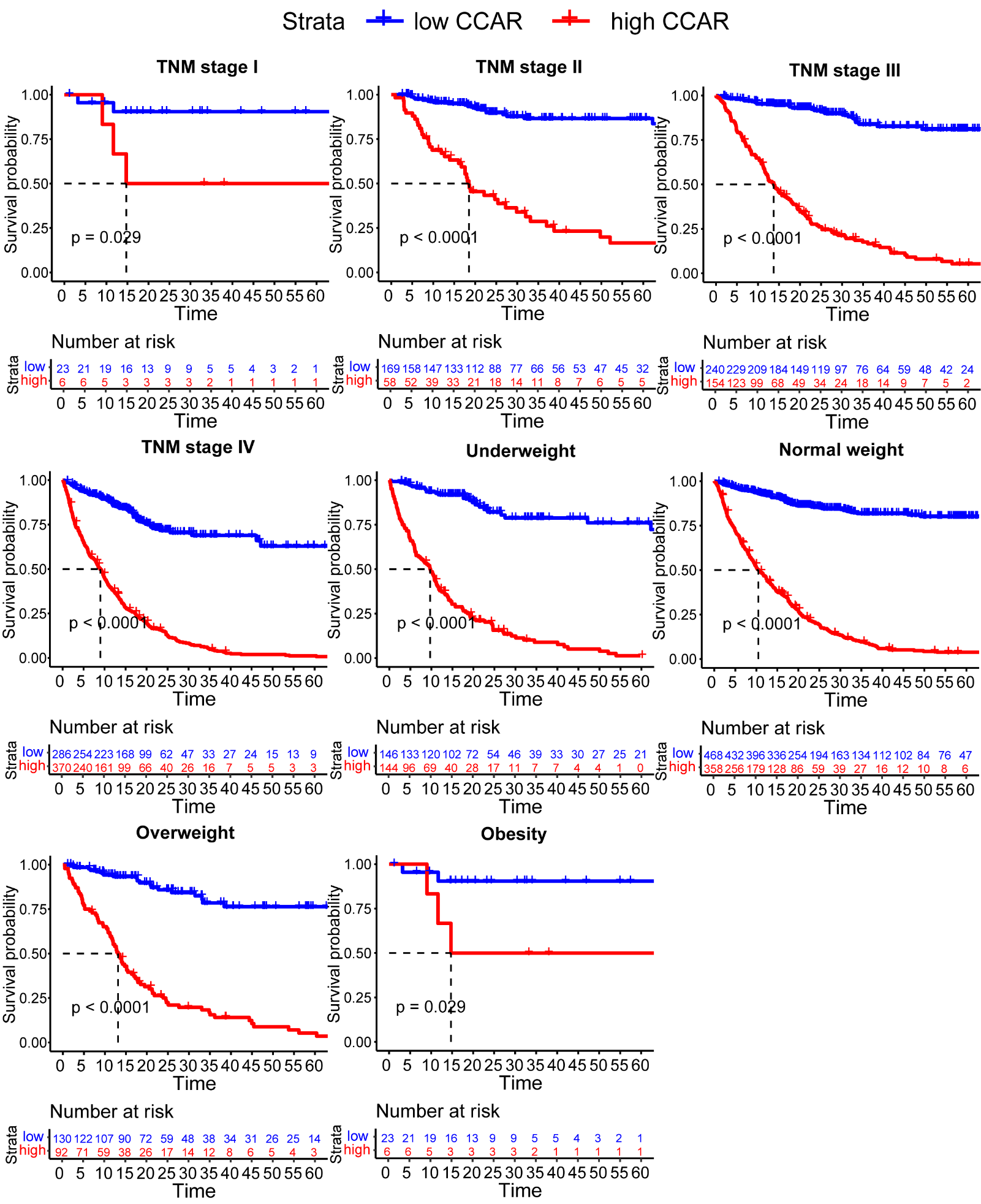


**Figure S9. Cancer type-specific stratified survival curves based on CCAR.**


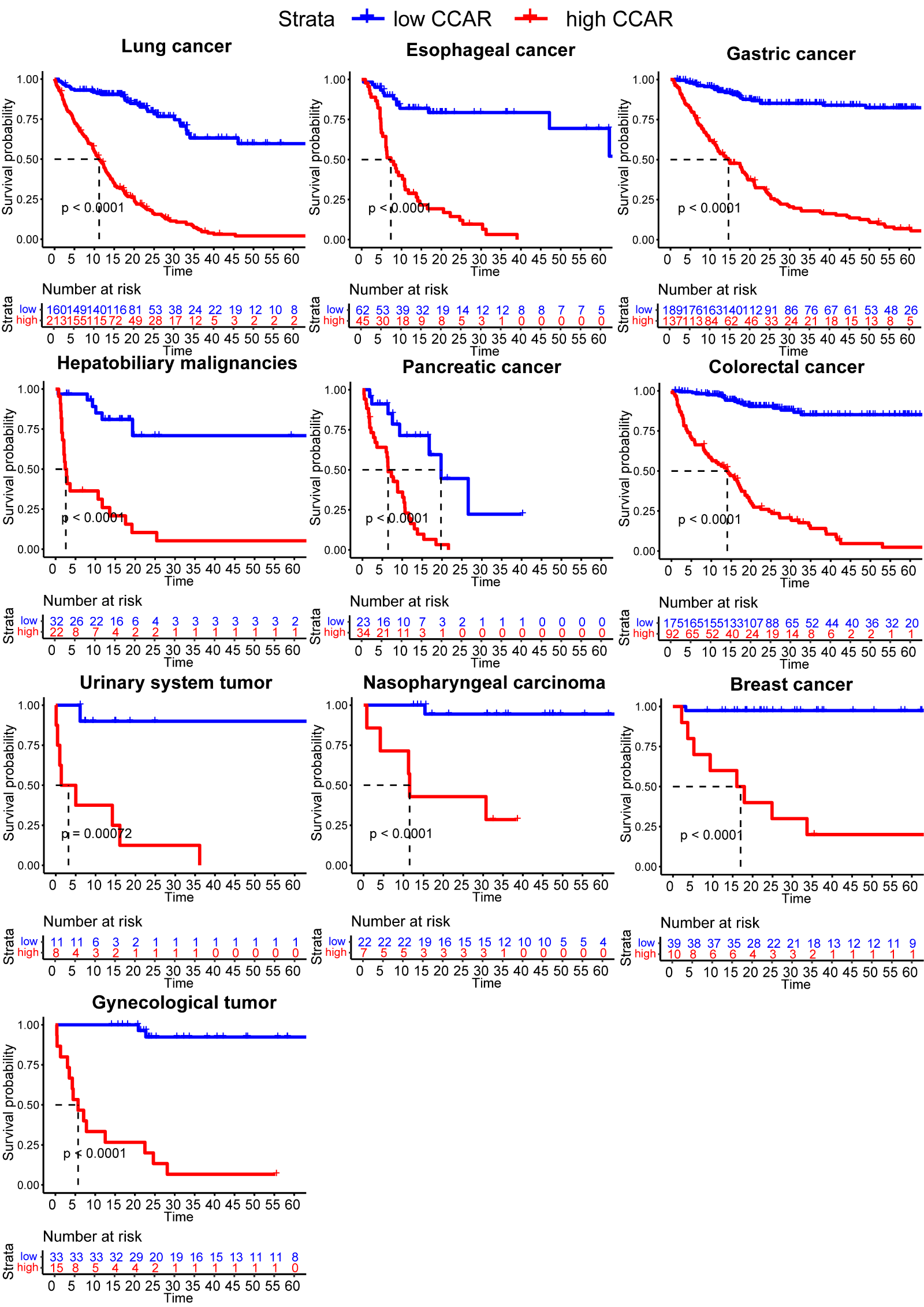


**Figure S10. Log-hazard ratio of CCAR for cancer cachexia prognosis.**


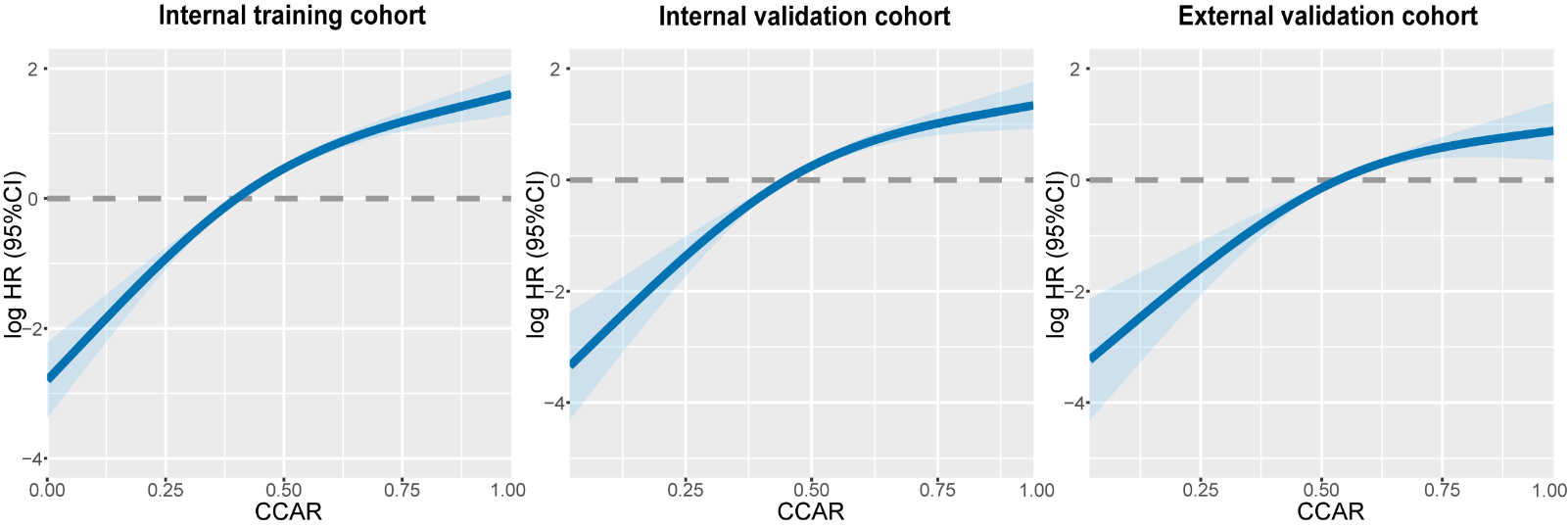


**Figure S11. Log-hazard ratio of CCAR for cancer cachexia prognosis in different model.**


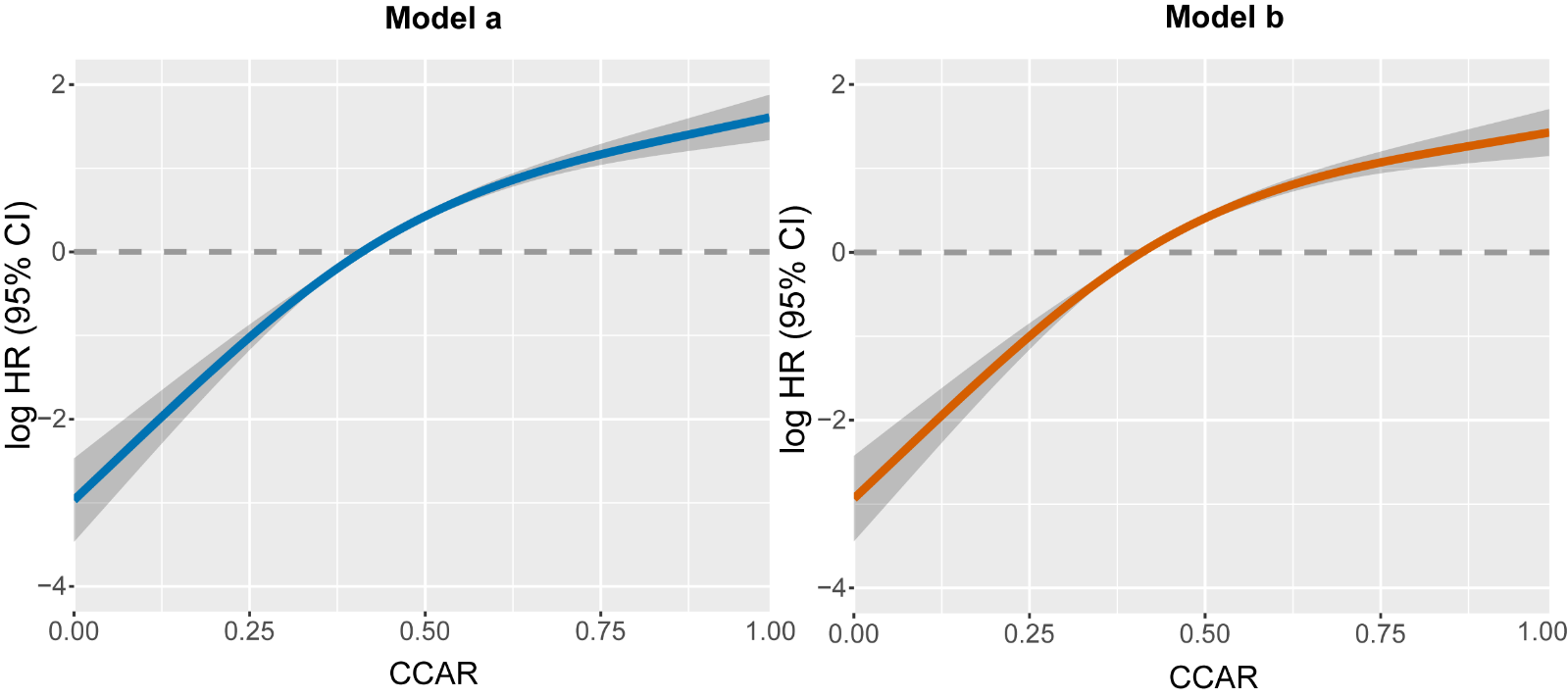


Notes:

Model a: No adjusted.

Model b: Adjusted for age, gender, BMI, TNM stage, surgery, coronary heart disease, drinking, gender, family history, smoking, chemotherapy, radiotherapy, diabetes, hypertension.

**Figure S12. The distribution of CCAR index in tumor stage and BMI.**


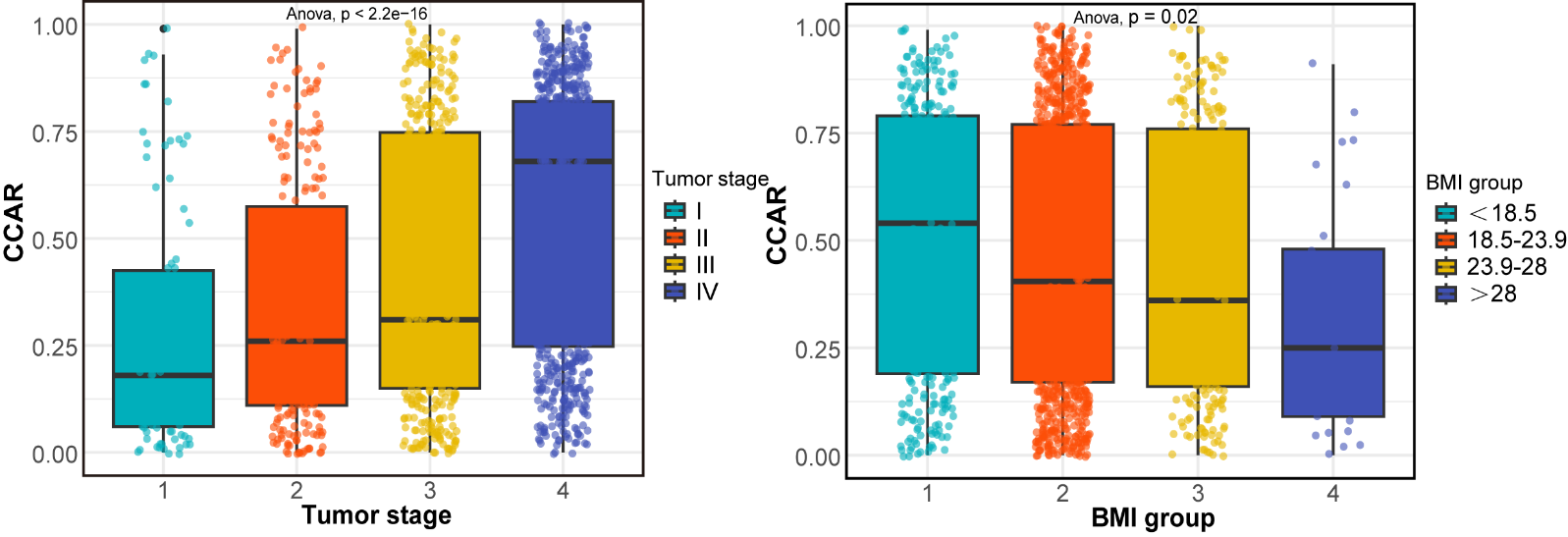


**Figure S13. The distribution of CCAR index in different tumor type.**


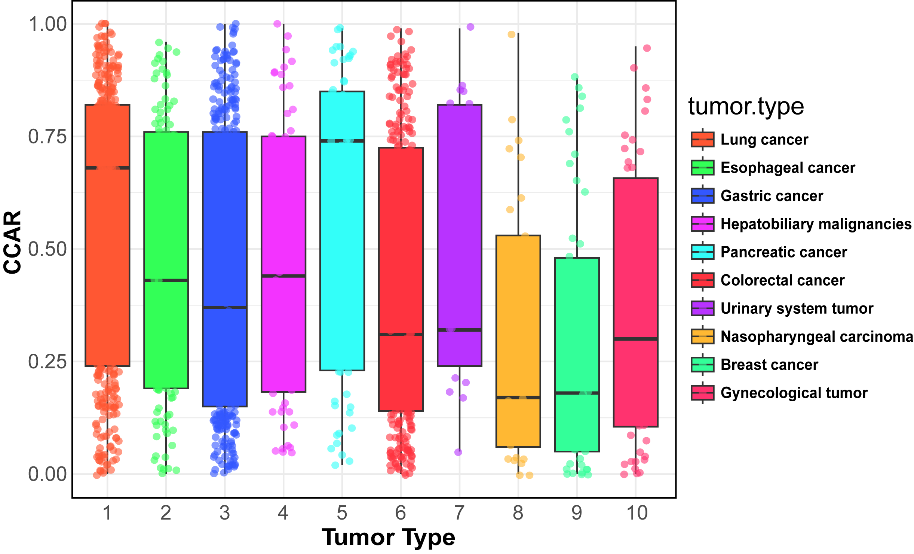


**Supplementary tables**

**Table S1. Inflammatory biomarkers evaluated in this study.**

| Biomarker name | Biomarker formulas |
| --- | --- |
| Neutrophil-to-Lymphocyte ratio (NLR) | Neutrophil (/µL) / lymphocyte (/µL) |
| Platelet-to-Lymphocyte ratio (PLR) | Platelet (/µL) / lymphocyte (/µL) |
| C-reactive protein-to-albumin ratio (CAR) | C-reactive protein (mg/dL) / albumin (g/dL) |
| Lymphocyte-to-C-reactive protein ratio (LCR) | Lymphocyte (/µL) / C-reactive protein (mg/dL) |
| Systemic-Immune-Inflammation Index (SII) | Platelet (/µL) × Neutrophil (/µL) / lymphocyte (/µL) |
| Prognostic Nutritional Index (PNI) | 10 × Albumin (g/dL) + 0.005 × lymphocyte (/µL) |
| Triglyceride to HDL ratio (TG.HDL) | Triglyceride (mg/dL) / HDL (mg/dL) |
| Total cholesterol to HDL ratio (TC.HDL) | Total cholesterol (mg/dL) / HDL (mg/dL) |
| Modified Glasgow Prognostic Score (mGPS) | 0: C-reactive protein ≤10 mg/dL and Albumin ≥3 g/dL  1: C-reactive protein ≤10 mg/dL and Albumin <3 g/dL  2: C-reactive protein >10 mg/dL |
| Modified Geriatric Nutritional Risk Index (mGNRI) | 14.89 × Albumin (g/dL) + 41.7 × (Body Mass Index - 18.5) |

**Table S2.** **Demographic and clinical characteristics of the study population**

| **Variable** | **Overall (n =1367)** |
| --- | --- |
| **Gender, Male (%)** | 855(62.5) |
| **Age, years, median (IQR)** | 59.00(52.00,67.00) |
| **Smoking, yes, n (%)** | 669(48.9) |
| **Drinking, yes, n (%)** | 334(24.4) |
| **Chemotherapy, yes, n (%)** | 850(62.2) |
| **Radiotherapy, yes, n (%)** | 99(7.2) |
| **Diabetes, yes, n (%)** | 135(9.9) |
| **Hypertension, yes, n (%)** | 237(17.3) |
| **Coronary disease, yes, n (%)** | 62(4.5) |
| **Family history, yes, n (%)** | 203(14.9) |
| **Surgery, yes, n (%)** | 200(14.6) |
| **BMI group (%)** |  |
| Underweight | 379(23.0) |
| Normal weight | 1087(65.8) |
| Overweight | 168(10.2) |
| Obesity | 17(1.0) |
| **Tumor stage, n (%)** |  |
| I | 90(6.6) |
| II | 227(16.6) |
| III | 394(28.8) |
| IV | 656(48.0) |
| **Tumor type, n (%)** |  |
| Lung cancer | 373(27.3) |
| Esophageal cancer | 107(7.8) |
| Gastric cancer | 326(23.8) |
| Hepatobiliary malignancies | 54(4.0) |
| Pancreatic cancer | 57(4.2) |
| Colorectal cancer | 267(19.5) |
| Urinary system tumor | 19(1.4) |
| Nasopharyngeal carcinoma | 29(2.1) |
| Breast cancer | 49(3.6) |
| Gynecological tumor | 48(3.5) |
| Others | 38(2.8) |
| **Total Protein, g/L, median (IQR)** | 67.50(62.50,72.00) |
| **Albumin, g/L, median (IQR)** | 37.90(34.20,41.20) |
| **CRP, mg/L, median (IQR)** | 5.99(3.08,27.10) |
| **Hb, g/L, median (IQR)** | 121.00(105.00,136.00) |
| **WBC, 10^9/L, median (IQR)** | 6.00(4.66,8.05) |
| **Neutrophils, 10^9/L, median (IQR)** | 3.80(2.48,5.64) |
| **Lymphocyte, 10^9/L, median (IQR)** | 1.45(1.03,1.85) |
| **RBC, 10^12/L, median (IQR)** | 4.17(3.69,4.57) |
| **PLT, 10^9/L, median (IQR)** | 234.00(178.00,302.00) |
| **HDL, mmol/L, median (IQR)** | 1.14(0.93,1.38) |
| **LDL, mmol/L, median (IQR)** | 2.66(2.18,3.18) |
| **TC, mmol/L, median (IQR)** | 4.37(3.71,5.04) |
| **TG, mmol/L, median (IQR)** | 1.16(0.88,1.54) |
| **LCR** | 0.21(0.05,0.52) |
| **CAR** | 0.16(0.08,0.74) |
| **NLR** | 2.63(1.66,4.31) |
| **PLR** | 164.66(115.03,237.21) |
| **PNI** | 379.01(342.00,412.01) |
| **SII** | 615.96(334.06,1123.92) |
| **TG.HDL.C** | 1.04(0.72,1.55) |
| **TC.HDL.C** | 3.80(3.13,4.64) |
| **mGPS** | 1.00(0.00,1.00) |
| **mGNRI** | 53.56(49.37,58.27) |

Abbreviations: Total Protein, Total protein; Albumin, Albumin; CRP, C-reactive protein; Hb, Hemoglobin; WBC, White blood cell; RBC, Red blood cell; PLT, Platelet; HDL, High-density lipoprotein; LDL, Low-density lipoprotein; TC, Total cholesterol; TG, Triglycerides; KPS, Karnofsky performance status; LCR, Lymphocyte-to-C-reactive protein ratio; CAR, C-reactive protein-to-albumin ratio; NLR, Neutrophil-to-lymphocyte ratio; PLR, Platelet-to-lymphocyte ratio; PNI, Prognostic nutritional index; SII, Systemic immune-inflammation index; TG.HDL.C, Triglyceride-to-high-density lipoprotein cholesterol ratio; TC.HDL.C, Total cholesterol-to-high-density lipoprotein cholesterol ratio; mGPS, Modified Glasgow prognostic score; mGNRI, Modified geriatric nutritional risk index.

**Table S3. Comparison of demographic and clinicopathological characteristics across different cohorts.**

| **Variable** | **Internal training**  **cohort (n =959)** | **Internal validation**  **cohort (n = 408)** | **External validation cohort (n = 291)** |
| --- | --- | --- | --- |
| **Gender, Male (%)** | 606(63.2) | 249(61.0) | 171(60.2) |
| **Age, years, median (IQR)** | 59.00(52.00,66.00) | 59.00(52.00,67.25) | 62.00(54.00,68.00) |
| **Smoking, yes, n (%)** | 476(49.6) | 193(47.3) | 108(38.0) |
| **Drinking, yes, n (%)** | 239(24.9) | 95(23.3) | 81(28.5) |
| **Chemotherapy, yes, n (%)** | 590(61.5) | 260(63.7) | 195(68.7) |
| **Radiotherapy, yes, n (%)** | 67(7.0) | 32(7.8) | 7(2.5) |
| **Diabetes, yes, n (%)** | 98(10.2) | 37(9.1) | 27(9.5) |
| **Hypertension, yes, n (%)** | 154(16.1) | 83(20.3) | 74(26.1) |
| **Coronary disease, yes, n (%)** | 44(4.6) | 18(4.4) | 5(1.8) |
| **Family history, yes, n (%)** | 133(13.9) | 70(17.2) | 67(23.6) |
| **Surgery, yes, n (%)** | 133(13.9) | 67(16.4) | 1(0.4) |
| **BMI group (%)** |  |  |  |
| Underweight | 219(22.8) | 71(17.4) | 89(31.3) |
| Normal weight | 578(60.3) | 248(60.8) | 161(56.7) |
| Overweight | 144(15.0) | 78(19.1) | 33(11.6) |
| Obesity | 18(1.9) | 11(2.7) | 1(0.4) |
| **Tumor stage, n (%)** |  |  |  |
| I | 57(5.9) | 33(8.1) | 6(2.1) |
| II | 160(16.7) | 67(16.4) | 21(7.4) |
| III | 273(28.5) | 121(29.7) | 80(28.2) |
| IV | 469(48.9) | 187(45.8) | 177(62.3) |
| **Tumor type, n (%)** |  |  |  |
| Lung cancer | 256(26.7) | 117(28.7) | 69(24.3) |
| Esophageal cancer | 81(8.4) | 26(6.4) | 21(7.4) |
| Gastric cancer | 226(23.6) | 100(24.5) | 68(23.9) |
| Hepatobiliary malignancies | 38(4.0) | 16(3.9) | 16(5.6) |
| Pancreatic cancer | 40(4.2) | 17(4.2) | 9(3.2) |
| Colorectal cancer | 188(19.6) | 79(19.4) | 66(23.2) |
| Urinary system tumor | 16(1.7) | 3(0.7) | 2(0.7) |
| Nasopharyngeal carcinoma | 19(2.0) | 10(2.5) | 2(0.7) |
| Breast cancer | 33(3.4) | 16(3.9) | 5(1.8) |
| Gynecological tumor | 33(3.4) | 15(3.7) | 9(3.2) |
| Others | 29(3.0) | 9(2.2) | 17(6.0) |
| **Follow-up duration, months (IQR)** | 16.67 (7.97, 28.82) | 16.67 (8.36, 30.17) | 20.30 (10.99, 27.33) |
| **Total Protein, g/L, median (IQR)** | 67.50 (62.40, 72.30) | 67.50 (63.08, 71.50) | 70.60 (65.97, 76.03) |
| **Albumin, g/L, median (IQR)** | 37.90 (34.00, 41.10) | 38.00 (34.50, 41.30) | 39.60 (35.45, 43.23) |
| **CRP, mg/L, median (IQR)** | 5.87 (3.10, 26.93) | 6.81 (3.02, 27.47) | 6.14 (1.60, 33.78) |
| **Hb, g/L, median (IQR)** | 121.00 (105.00, 136.00) | 121.50 (106.75, 135.00) | 110.50 (81.75, 127.00) |
| **WBC, 10^9/L, median (IQR)** | 6.00 (4.59, 8.14) | 6.02 (4.80, 7.91) | 5.90 (4.40, 7.93) |
| **Neutrophils, 10^9/L, median (IQR)** | 3.81 (2.42, 5.74) | 3.78 (2.64, 5.47) | 4.00 (2.80, 6.30) |
| **Lymphocyte, 10^9/L, median (IQR)** | 1.43 (1.02, 1.84) | 1.46 (1.05, 1.87) | 1.10 (0.80, 1.60) |
| **RBC, 10^12/L, median (IQR)** | 4.16 (3.68, 4.56) | 4.20 (3.73, 4.61) | 3.84 (3.35, 4.26) |
| **PLT, 10^9/L, median (IQR)** | 234.00 (176.00, 301.00) | 232.00 (182.75, 305.25) | 219.00 (155.75, 287.00) |
| **HDL, mmol/L, median (IQR)** | 1.16 (0.95, 1.38) | 1.13 (0.90, 1.36) | 1.01 (0.84, 1.23) |
| **LDL, mmol/L, median (IQR)** | 2.66 (2.17, 3.20) | 2.67 (2.20, 3.16) | 2.47 (2.04, 2.98) |
| **TC, mmol/L, median (IQR)** | 4.38 (3.70, 5.06) | 4.32 (3.74, 5.01) | 4.22 (3.63, 4.80) |
| **TG, mmol/L, median (IQR)** | 1.15 (0.88, 1.52) | 1.20 (0.88, 1.61) | 1.12 (0.84, 1.62) |
| **LCR** | 0.22 (0.05, 0.52) | 0.21 (0.05, 0.53) | 0.20 (0.03, 0.76) |
| **CAR** | 0.16 (0.08, 0.74) | 0.17 (0.07, 0.73) | 0.15 (0.04, 0.91) |
| **NLR** | 2.70 (1.66, 4.36) | 2.48 (1.64, 4.16) | 3.65 (2.18, 6.26) |
| **PLR** | 163.66 (114.88, 238.56) | 166.87 (117.32, 231.89) | 195.42 (133.48, 279.00) |
| **PNI** | 379.01 (340.01, 411.01) | 380.01 (345.01, 413.01) | 396.01 (354.50, 432.25) |
| **SII** | 618.88 (333.63, 1161.53) | 610.49 (334.34, 1064.90) | 760.50 (433.11, 1629.94) |
| **TG.HDL.C** | 1.01 (0.72, 1.51) | 1.10 (0.74, 1.63) | 1.12 (0.77, 1.77) |
| **TC.HDL.C** | 3.77 (3.09, 4.64) | 3.85 (3.24, 4.64) | 4.03 (3.34, 4.96) |
| **mGPS** | 1.00 (0.00, 1.00) | 1.00 (0.00, 1.00) | 0.00 (0.00, 1.00) |
| **mGNRI** | 53.20 (49.11, 57.98) | 54.41 (50.43, 58.94) | 52.96 (48.57, 57.50) |
| **CCAR** | 0.40 (0.16, 0.77) | 0.45 (0.18, 0.79) | 0.54 (0.16, 0.83) |

Abbreviations: Total Protein, Total protein; Albumin, Albumin; CRP, C-reactive protein; Hb, Hemoglobin; WBC, White blood cell; RBC, Red blood cell; PLT, Platelet; HDL, High-density lipoprotein; LDL, Low-density lipoprotein; TC, Total cholesterol; TG, Triglycerides; KPS, Karnofsky performance status; LCR, Lymphocyte-to-C-reactive protein ratio; CAR, C-reactive protein-to-albumin ratio; NLR, Neutrophil-to-lymphocyte ratio; PLR, Platelet-to-lymphocyte ratio; PNI, Prognostic nutritional index; SII, Systemic immune-inflammation index; TG.HDL.C, Triglyceride-to-high-density lipoprotein cholesterol ratio; TC.HDL.C, Total cholesterol-to-high-density lipoprotein cholesterol ratio; mGPS, Modified Glasgow prognostic score; mGNRI, Modified geriatric nutritional risk index.

**Table S4. C-index values of various biomarkers in different cohorts.**

| **Internal training cohort** | | | | **Internal validation cohort** | | | | | **External validation cohort** | | | | |
| --- | --- | --- | --- | --- | --- | --- | --- | --- | --- | --- | --- | --- | --- |
|  | **c-index** | **lower.95** | **upper.95** |  | **c-index** | | **lower.95** | **upper.95** |  | **c-index** | | **lower.95** | **upper.95** |
| **CAR** | 0.627 | 0.599 | 0.654 | **CAR** | | 0.656 | 0.617 | 0.694 | **CAR** | | 0.660 | 0.613 | 0.708 |
| **NLR** | 0.625 | 0.597 | 0.652 | **LCR** | | 0.654 | 0.616 | 0.692 | **LCR** | | 0.660 | 0.613 | 0.707 |
| **LCR** | 0.621 | 0.593 | 0.648 | **NLR** | | 0.645 | 0.607 | 0.683 | **NLR** | | 0.621 | 0.570 | 0.672 |
| **mGPS** | 0.602 | 0.577 | 0.628 | **mGPS** | | 0.630 | 0.596 | 0.665 | **SII** | | 0.606 | 0.554 | 0.657 |
| **PNI** | 0.601 | 0.573 | 0.630 | **SII** | | 0.621 | 0.583 | 0.659 | **mGPS** | | 0.601 | 0.554 | 0.647 |
| **SII** | 0.601 | 0.573 | 0.629 | **mGNRI** | | 0.580 | 0.541 | 0.620 | **PNI** | | 0.598 | 0.549 | 0.648 |
| **mGNRI** | 0.570 | 0.543 | 0.598 | **PLR** | | 0.552 | 0.512 | 0.592 | **mGNRI** | | 0.585 | 0.534 | 0.635 |
| **TG.HDL** | 0.552 | 0.523 | 0.580 | **TG.HDL** | | 0.543 | 0.502 | 0.584 | **PLR** | | 0.543 | 0.490 | 0.596 |
| **TC.HDL** | 0.545 | 0.516 | 0.574 | **TC.HDL** | | 0.522 | 0.482 | 0.561 | **TG.HDL** | | 0.504 | 0.450 | 0.557 |
| **PLR** | 0.533 | 0.503 | 0.563 | **PNI** | | 0.396 | 0.357 | 0.436 | **TC.HDL** | | 0.485 | 0.432 | 0.538 |

**Table S5. C-index values of CCAR, CAR, NLR, LCR, mGPS, PNI, and mGNRI in different cohorts.**

| **Internal training cohort** | | | | **Internal validation cohort** | | | | | **External validation cohort** | | | | |
| --- | --- | --- | --- | --- | --- | --- | --- | --- | --- | --- | --- | --- | --- |
|  | **c-index** | **lower.95** | **upper.95** |  | **c-index** | | **lower.95** | **upper.95** |  | **c-index** | | **lower.95** | **upper.95** |
| **CCAR** | 0.777 | 0.757 | 0.798 | **CCAR** | | 0.789 | 0.757 | 0.820 | **CCAR** | | 0.765 | 0.728 | 0.803 |
| **CAR** | 0.627 | 0.599 | 0.654 | **CAR** | | 0.649 | 0.607 | 0.692 | **CAR** | | 0.660 | 0.613 | 0.708 |
| **NLR** | 0.625 | 0.597 | 0.652 | **LCR** | | 0.646 | 0.603 | 0.689 | **LCR** | | 0.660 | 0.613 | 0.707 |
| **LCR** | 0.621 | 0.593 | 0.648 | **PNI** | | 0.643 | 0.602 | 0.685 | **NLR** | | 0.621 | 0.570 | 0.672 |
| **mGPS** | 0.602 | 0.577 | 0.628 | **SII** | | 0.637 | 0.596 | 0.678 | **SII** | | 0.606 | 0.554 | 0.657 |
| **PNI** | 0.601 | 0.573 | 0.63 | **mGPS** | | 0.632 | 0.595 | 0.669 | **mGPS** | | 0.601 | 0.554 | 0.647 |
| **SII** | 0.601 | 0.573 | 0.629 | **NLR** | | 0.630 | 0.588 | 0.671 | **PNI** | | 0.598 | 0.549 | 0.648 |
| **mGNRI** | 0.57 | 0.543 | 0.598 | **mGNRI** | | 0.579 | 0.534 | 0.624 | **mGNRI** | | 0.585 | 0.534 | 0.635 |
| **TG.HDL** | 0.552 | 0.523 | 0.58 | **PLR** | | 0.578 | 0.536 | 0.620 | **PLR** | | 0.543 | 0.490 | 0.596 |
| **TC.HDL** | 0.545 | 0.516 | 0.574 | **TG.HDL** | | 0.544 | 0.499 | 0.588 | **TG.HDL** | | 0.504 | 0.450 | 0.557 |
| **PLR** | 0.533 | 0.503 | 0.563 | **TC.HDL** | | 0.541 | 0.494 | 0.587 | **TC.HDL** | | 0.485 | 0.432 | 0.538 |

**Table S6. Comparison of the prediction ability among different models through NRI and IDI.**

| **Variable** | **NRI (95% CI)** | ***P*NRI** | **IDI (95% CI)** | ***P*NRI** |
| --- | --- | --- | --- | --- |
| **CCAR vs. TNM stage** | 0.490 (0.416, 0.564) | <0.001 | 0.269 (0.252, 0.287) | <0.001 |
| **CCAR vs. BMI** | 1.171 (1.091, 1.250) | <0.001 | 0.410 (0.393, 0.428) | <0.001 |

NRI, net reclassification index; IDI, integrated discrimination improvement
